# Sensitivity optimization of a rhodopsin-based fluorescent voltage indicator

**DOI:** 10.1016/j.neuron.2023.03.009

**Published:** 2023-04-03

**Authors:** Ahmed S Abdelfattah, Jihong Zheng, Amrita Singh, Yi-Chieh Huang, Daniel Reep, Getahun Tsegaye, Arthur Tsang, Benjamin J Arthur, Monika Rehorova, Carl VL Olson, Yichun Shuai, Lixia Zhang, Tian-Ming Fu, Daniel E Milkie, Maria V Moya, Timothy D Weber, Andrew L Lemire, Christopher A Baker, Natalie Falco, Qinsi Zheng, Jonathan B Grimm, Mighten C Yip, Deepika Walpita, Martin Chase, Luke Campagnola, Gabe J Murphy, Allan M Wong, Craig R Forest, Jerome Mertz, Michael N Economo, Glenn C Turner, Minoru Koyama, Bei-Jung Lin, Eric Betzig, Ondrej Novak, Luke D Lavis, Karel Svoboda, Wyatt Korff, Tsai-Wen Chen, Eric R Schreiter, Jeremy P Hasseman, Ilya Kolb

**Affiliations:** 1 Janelia Research Campus, Howard Hughes Medical Institute, Ashburn, VA, USA; 2 GENIE Project Team, Janelia Research Campus, Howard Hughes Medical Institute, Ashburn, VA, USA; 3 Institute of Neuroscience, National Yang Ming Chiao Tung University, Taipei, Taiwan; 4 Department of Physiology, Second Faculty of Medicine, Charles University, Prague, Czech Republic; 5 Department of Biomedical Engineering, Boston University, Boston, MA, USA; 6 Allen Institute for Brain Science, Seattle, WA, USA; 7 George W. Woodruff School of Mechanical Engineering, Georgia Institute of Technology, Atlanta, GA, USA; 8 Unaffiliated; 9 Departments of Molecular and Cell Biology and Physics, Howard Hughes Medical Institute, Helen Wills Neuroscience Institute, University of California, Berkeley, Berkeley, CA, USA; 10 Molecular Biophysics and Integrated Bioimaging Division, Lawrence Berkeley National Laboratory, Berkeley, CA, USA; 11 Present address: Department of Neuroscience, Brown University, Providence, RI, USA; 12 Present address: Carney Institute for Brain Science, Brown University, Providence, RI, USA; 13 Present address: National Institutes of Health, Bethesda, MD, USA; 14 Present address: Department of Neurology, Yale School of Medicine, New Haven, CT, USA; 15 Present address: Center for Neuroscience and Regeneration Research, Yale University, New Haven, CT, USA; 16 Present address: Rehabilitation Research Center, Veterans Affairs Connecticut Healthcare System, West Haven, CT, USA; 17 Present address: Department of Biological Sciences, University of Toronto Scarborough, Toronto, Canada; 18 Present address: Department of Cell and Systems Biology, University of Toronto, Toronto, Canada; 19 Present address: Allen Institute for Neural Dynamics, Seattle, WA, 98109, USA; 20 Lead contact

## Abstract

The ability to optically image cellular transmembrane voltage at millisecond-timescale resolution can offer unprecedented insight into the function of living brains in behaving animals. Here, we present a point mutation that increases the sensitivity of Ace2 opsin-based voltage indicators. We use the mutation to develop Voltron2, an improved chemigeneic voltage indicator which has a 65% higher sensitivity to single APs and 3-fold higher sensitivity to subthreshold potentials than Voltron. Voltron2 retained the sub-millisecond kinetics and photostability of its predecessor, although with lower baseline fluorescence. In multiple *in vitro* and *in vivo* comparisons with its predecessor across multiple species, we found Voltron2 to be more sensitive to APs and subthreshold fluctuations. Finally, we used Voltron2 to study and evaluate possible mechanisms of interneuron synchronization in the mouse hippocampus. Overall, we have discovered a generalizable mutation that significantly increases the sensitivity of Ace2 rhodopsin-based sensors, improving their voltage reporting capability.

## eTOC blurb

Abdelfattah et al. describe a point mutation that increases the sensitivity of voltage indicators. A new voltage indicator carrying that mutation (Voltron2) is found to have improved sensitivity over its predecessor. Voltron2 is validated *in vivo* and used to study the subthreshold activity of interneurons in the living mouse brain.

## Introduction

Genetically encoded voltage indicators (GEVIs) have served as an enabling technology for visualizing neuronal activity at unprecedented spatiotemporal resolution^1,2^. Nevertheless, optical imaging of voltage using GEVIs presents many challenges for the design of these proteins. An ideal voltage sensor must concurrently fulfill many requirements, including but not limited to: (1) high sensitivity to membrane potential changes of a neuron, (2) fluorescence changes that are fast enough to follow and accurately report APs and (3) high degree of localization to the cell surface ^3,4^. Further requirements may be desirable depending on application, such as sensitivity to sub-threshold membrane potential changes, photostability, and compatibility with two-photon excitation ^5^.

One approach to engineering GEVIs involves exploiting the native voltage sensitivity of microbial rhodopsins. The opsin Archaerhodopsin 3 (Arch) was first successfully used to optically record APs in neuronal culture^6^; however, it was found to be too dim at physiologically tolerable imaging powers for *in vivo* applications. Subsequent protein engineering efforts of Arch yielded improvements in brightness as well as sensitivity, kinetics, and reduced photocurrents^1,7–11^. An alternative strategy to develop bright rhodopsin-based GEVIs was to create a Förster resonance energy transfer (FRET) pair between a bright fluorescent protein (FP) and the rhodopsin protein^12,13^. In this strategy, the bright FP is the reporter fluorophore, and the rhodopsin is used as the voltage sensitive domain. This strategy was successfully implemented to develop Ace2N-mNeon, a bright fast GEVI that was able to report single APs *in vivo*^12^.

The Ace2N-mNeon member of the rhodopsin family of GEVIs has been used as a scaffold to create GEVIs with other favorable characteristics. For example, a red indicator called VARNAM consisting of Ace2N fused to a red FP mRuby3 displayed high sensitivity, good *in vivo* performance, and a spectral shift that made it compatible with blue-shifted optogenetic probes^14^. Recently, improved and positive-going versions of these indicators have been developed^15^. Our group also previously used the Ace2N-mNeon scaffold, replacing the FP with a HaloTag protein^16^ covalently bound to a small-molecule fluorophore (JaneliaFluor or JF^17,18^) to create a chemigenetic indicator called Voltron^19^. The introduction of three point mutations to the rhodopsin domain of Voltron led to Positron, a positive-going indicator with sensitivity and kinetics comparable to the original Voltron^20^.

Encouraged by the ability of point mutations in the rhodopsin domain to alter function, in this study, we performed a large-scale screen of point mutations to find improved versions of Voltron. We discovered that the introduction of an A122D mutation increased the sensitivity of Voltron, particularly in the sub-threshold range, without compromising kinetics, membrane trafficking or photobleaching. Thus Voltron.A122D was named Voltron2 as a next-generation version of the sensor. Consistent with the observation in culture, *in vivo* imaging in flies, zebrafish and mice revealed an increased signal-to-noise ratio (SNR) of Voltron2 compared to Voltron.

## Results

### High throughput screening of Voltron mutants

**Figure 1:**
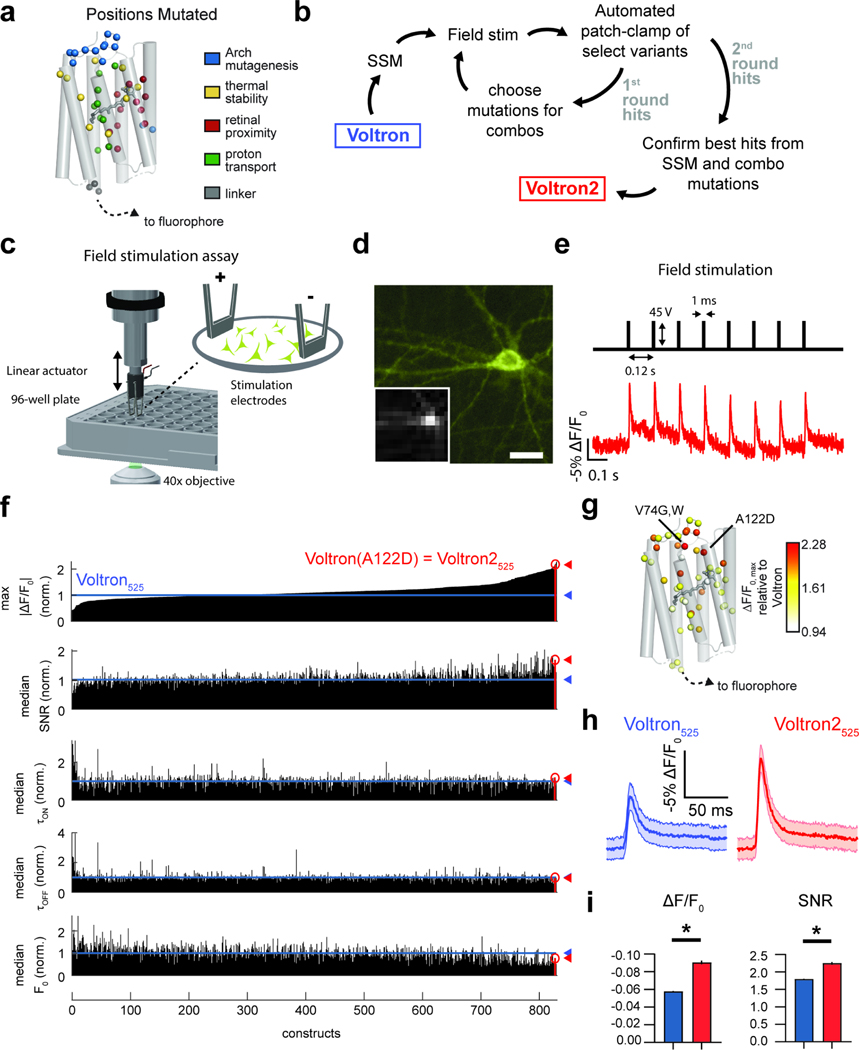
Mutagenesis and screening of Voltron in cultured neurons. a. Residues targeted for SSM in the Ace2N rhodopsin domain of Voltron, colored by the rationale for targeting them. b. Mutagenesis and screening workflow. c. Diagram of field stimulation assay performed in 96-well plates. d. Representative image of a neuron from the screen expressing Voltron2_525_. Inset shows representative frame during fast (1.497 kHz) stream acquisition. Scale bar: 10 μm. e. Field stimulation parameters (top, black) and acquired fluorescence response of the neuron shown in d (bottom, red). All imaging in the screen was performed at a light density of 1.14 mW/mm^2^ measured in the image plane. f. Field stimulation assay results of the SSM Voltron_525_ screen, ranked by maximum $\left|\text{ΔF}/{\text{F}}_{0}\right|$ for each variant, normalized to in-plate Voltron_525_ controls. g. Mutated residues colored by the maximum increase in $\left|\text{ΔF}/{\text{F}}_{0}\right|$ achieved in that position. Top three mutations are labeled. h. Representative traces (mean ± s.e.m.) from a single plate containing Voltron_525_ (8 wells) and Voltron2_525_ (8 wells). i. Single AP $\text{ΔF}/{\text{F}}_{0}$ (mean ± s.e.m.; Voltron_525_, −.059 ± 0.001, n=338 wells; Voltron2_525_: −0.090 ± 0.002, n=130 wells; p<0.0001, Mann-Whitney U test) and SNR of Voltron2_525_ and SNR (Voltron_525_: 1.80 ± 0.013; Voltron2_525_: 2.24 ± 0.040; p<0.0001, Mann-Whitney U test). See also Fig. S1.

Voltron variants were generated using site saturation mutagenesis (SSM) performed at 40 positions within the rhodopsin domain. All screening was performed on Voltron mutants labeled with JF_525_ (referred to as Voltron_525_). Positions were chosen based on: (1) previous reports of analogous positions in other opsins that affected their thermal stability^10,21–24^, (2) amino acids in close proximity to the retinal chromophore that we reasoned might affect the environment of the Schiff base, or (3) positions that were found to be important in mutagenesis of Archaerhodopsin into a voltage sensor^1^ (Fig 1a). We performed two rounds of screening (Fig. 1b). In the first, we screened individual point mutations using a field stimulation assay in primary neurons (Fig. 1c-e). For each variant, parameters relevant to the performance of the sensor *in vivo* were measured: AP sensitivity $\left(\text{ΔF}/{\text{F}}_{0}\right)$, AP rise and decay kinetics ($\tau_{ON}$ and $\tau_{OFF}$), and baseline fluorescence $\left({\text{F}}_{0}\right)$. To control for biological variability, the measured parameters of each construct were also normalized to an in-plate Voltron_525_ control. The control was also used to monitor the quality and consistency of the screen. For a construct screened in a 96-well plate, results were discarded if at least one of the following quality control (QC) criteria (empirically determined) were violated: (1) the average $\left|\text{ΔF}/{\text{F}}_{0}\right|$ of the in-plate Voltron_525_ controls was < 3.6%, (2) the percent detectable improvement (PDI) of the plate was > 30% (see Methods), or (3) the construct had < 100 pixels with a significant change in $\text{ΔF}/{\text{F}}_{0}$ during the stimulation (“responsive pixels”).

Of the 2,727 variants that were screened in 199 plates, 2,314 (84%) passed the above QC criteria. Variants that failed QC were re-screened again and 34% of them passed QC on the second round of screening and were added to the main QC-passed pool. The majority (66%) of the libraries were then sequenced and results from the same mutation were grouped, resulting in 819 QC-passing mutants. We found 422 mutants (51%) with significantly improved $\text{ΔF}/{\text{F}}_{0}$, 310 mutants (38%) with increased SNR, 233 mutants (27%) with reduced $\tau_{ON}$, 256 mutants (31%) with reduced $\tau_{OFF}$, and 307 (37%) with increased ${\text{F}}_{0}$ compared to Voltron (Fig. 1f, p<0.01, Mann-Whitney U test). The key feature of Voltron we desired to optimize was $\text{ΔF}/{\text{F}}_{0}$; therefore, we ranked all variants based on ${\left|\text{ΔF}/{\text{F}}_{0}\right|}_{\max}$ (maximum of $\left|\text{ΔF}/{\text{F}}_{0}\right|$) normalized to in-plate Voltron controls.

Although many variants had improved ${\left|\text{ΔF}/{\text{F}}_{0}\right|}_{\max}$ over Voltron_525_, there was no single top-performing variant in this first round of screening. Instead, the difference in ${\left|\text{ΔF}/{\text{F}}_{0}\right|}_{\max}$ of the top 3 variants was only ~10%, which was lower than our PDI metric (14±5.2% mean±s.d. across the first screening round), indicating that the ranking of the top variants may not be accurate. The top two hits in the screen were Voltron_525_.V74G (${\left|\text{ΔF}/{\text{F}}_{0}\right|}_{\max}$ relative to Voltron_525_ = 2.28) and Voltron_525_.V74W (${\left|\text{ΔF}/{\text{F}}_{0}\right|}_{\max}$ relative to Voltron_525_ = 2.21; Fig. 1g, Table S1). However, subsequent analysis with patch-clamp revealed that Voltron_525_.A122D (3^rd^ in the ranked ${\left|\text{ΔF}/{\text{F}}_{0}\right|}_{\max}$ list, ${\left|\text{ΔF}/{\text{F}}_{0}\right|}_{\max}$ relative to Voltron_525_ = 2.18) had superior properties as a voltage sensor. The Voltron_525_.A122D mutant (which we named Voltron2_525_) exhibited ${\left|\text{ΔF}/{\text{F}}_{0}\right|}_{\max}$ and SNR that was 52% and 25%, respectively, higher than Voltron_525_ (Fig. 1 h,i).

The first-round SSM screen revealed many mutations that moderately increased $\text{ΔF}/{\text{F}}_{0}$. We therefore embarked on a second round of combinatorial (combo) screening, with the expectation that combining 13 of the top performing mutations (Y63L, N69E, V74E/W, R78H, N81S, L89A/C/G/T, A122D/H, V196P) would further improve the sensor. Of the 1,232 constructs screened in 106 plates of the combo screen, 77% passed QC. Surprisingly, only 28 of 848 combo mutants (3.3%) had significantly improved ${\left|\text{ΔF}/{\text{F}}_{0}\right|}_{\max}$ over Voltron2_525_ (p<0.01, Mann-Whitney U test; Fig. S1, Table S2). Similarly, only a few variants exhibited significantly increased SNR (20 of 848, 2.4%). The A122D substitution was present in 34% of the combo variants passing QC (Table S3); nevertheless, the combo screen revealed that combining it with other mutations resulted in less sensitive variants. Subsequent automated patch-clamp analysis confirmed that Voltron2_525_, containing the sole A122D substitution, outperformed all combo mutants (Fig. S4).

### Screening and characterization with automated whole-cell electrophysiology

Several single and combo mutation hits from the field stimulation assay had improved ${\left|\text{ΔF}/{\text{F}}_{0}\right|}_{\max}$ over Voltron but had very similar $\text{ΔF}/{\text{F}}_{0}$ characteristics among them. We deemed the field stimulation assay to be insufficiently sensitive to find the one variant with the best performance, so we used the uM Workstation, a fully automated whole-cell electrophysiology platform based on the PatcherBot^25^ to perform a secondary screen on top single and combinatorial mutant hits.

We first validated the throughput and performance of the automated electrophysiology platform. To mimic a small-scale screen, 10 35-mm Mattek dishes of cultured neurons were transfected with variants of the voltage sensor ASAP1^26^. The uM Workstation made 103 patch-clamp attempts in 7.1 hours, with a 78% whole-cell success rate. The system operated unattended for ~5 hours during that day of screening. Thus, the uM Workstation allowed us to screen ~10 constructs per day, assuming 5–10 neurons per construct.

**Figure 2:**
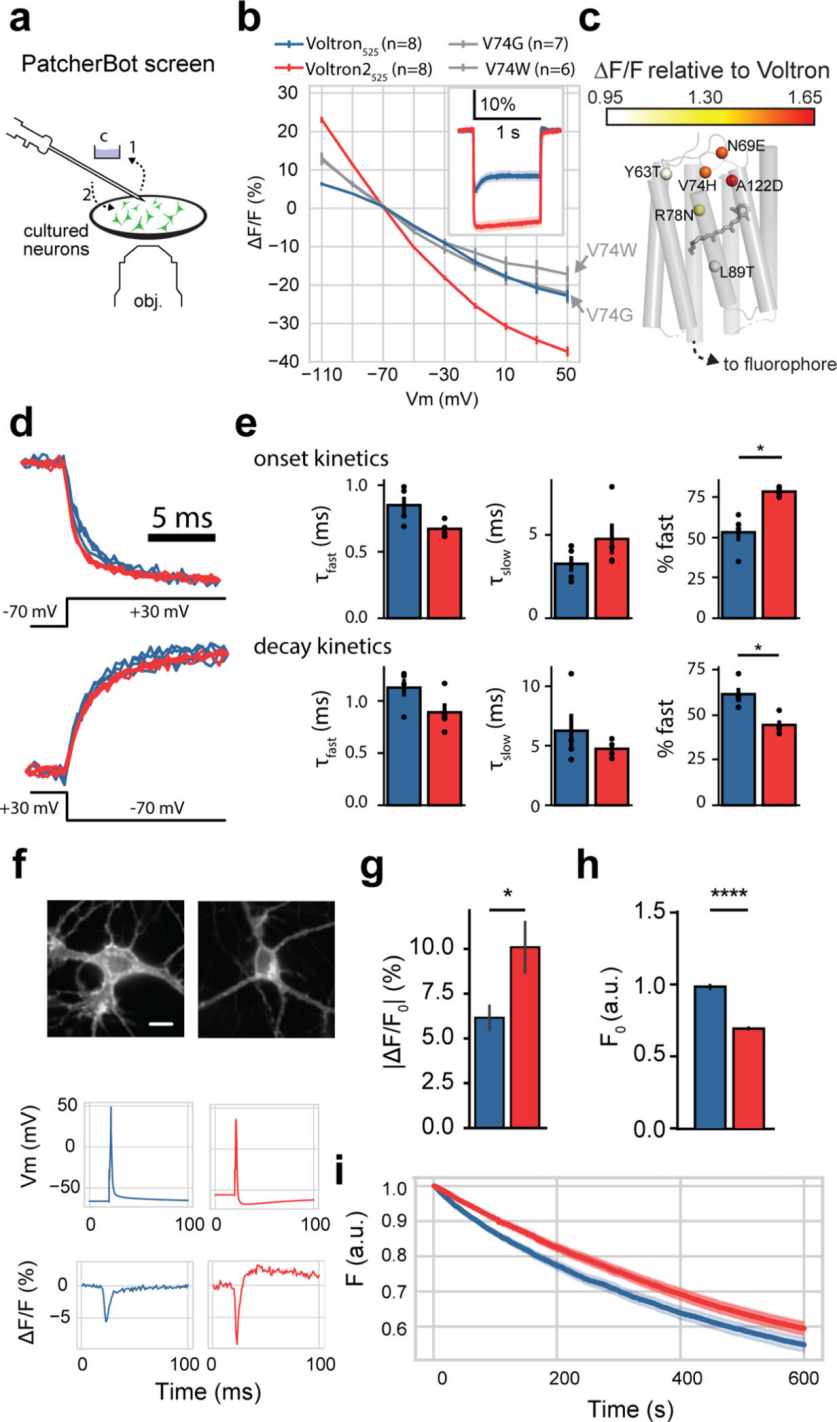
Automated patch-clamp screening and characterization of Voltron2 in cultured neurons. a. Fully automated uM workstation screening platform, based on PatcherBot. The pipette cleaning procedure is shown where a used pipette is dipped into a reservoir of cleaning solution (step 1, “c”) and back to the neuronal culture for a subsequent patch-clamp attempt without the need for replacing the pipette (step 2). b. Peak fluorescence responses to voltage steps (−70 to +30 mV) of Voltron_525_, Voltron2_525_ and the top two variants from the field stimulation assay (mean ± s.e.m.; Voltron2_525_ vs. Voltron_525_: p=0.012; Voltron2_525_ vs. Voltron_525_.V74G: p=0.015; Voltron2_525_ vs. Voltron_525_.V74W: p=0.0003, one-way ANOVA followed by Dunnett’s post-hoc test). Inset: Voltron_525_ and Voltron2_525_ fluorescence traces (solid line: mean, shading: s.e.m.) in response to −70 to +30 mV voltage steps. N values (cells) indicated in figure. c. Mutated residues from 1^st^ screening round (single sites) colored by the maximum $\text{ΔF}/{\text{F}}_{0}$ response to 100 mV (−70 to +30 mV) voltage steps, measured with the uM workstation. Top mutations at each position are labeled. d. Onset (top) and decay (bottom) fluorescence kinetics of Voltron_525_ and Voltron2_525_ in response to a +100 mV voltage step from −70 mV. Vertical axis scaled to match $\text{ΔF}/{\text{F}}_{0}$ between the sensors. e. Onset and decay kinetics (mean ± s.e.m.) of the traces in (d). Onset kinetics: *p=0.03, Mann-Whitney U test. Decay kinetics: *p=0.03, Mann-Whitney U test; Voltron_525_: n=4 cells, Voltron2_525_: n=4 cells. f. Representative fluorescence responses to single evoked APs in current clamp. Scale bar: 10 μm. g. $\text{ΔF}/{\text{F}}_{0}$ in response to single AP stimulation in current clamp mode (mean ± s.e.m.; *p=0.03, Student’s t test, Voltron_525_: n=5 cells, Voltron2_525_: n=7 cells). h. Normalized resting fluorescence relative to mTagBFP2 fused to the C terminus (mean ± s.e.m.; ****p<0.0001; Voltron_525_: n=105 cells, Voltron2_525_: n=115 cells, Student’s t test). i. Photobleaching comparison of Voltron_525_ and Voltron2_525_ over 10 mins (solid line: mean; shading: s.e.m.). All experiments were performed at room temperature. See also Figs. S2-S5.

The uM Workstation achieves high throughput by automatically cleaning and reusing patch-clamp pipettes (Fig. 2a); however, it is conceivable that the cleaning process is imperfect and whole-cell success rate degrades over subsequent attempts. To address this, we evaluated pipette performance after multiple patch-clamp attempts. Whole-cell success rate decreased over time, but likely due to cell health degradation, not due to an accumulation of debris on the reused pipette, since replacing the pipette did not recover the success rate (Fig. S2a). In a separate experiment we replaced the dish without replacing the pipette, and found that the success rate recovered, further suggesting that cell health degradation, not pipette debris is responsible for the apparent decrease in success rate (Fig. S2b). To explore the limits of pipette cleaning, we patch-clamped cells with the same pipette, replacing the plate as needed, until the time to form a GΩ seal increased, indicating a contaminated pipette. Consistent with previous observations, a single pipette could be used for patch-clamping ~50 neurons^25^ (Fig. S2c). Last, we evaluated the quality of the recordings and found 85.6% (143 out of 167) of the successful whole-cell recordings had a holding current greater than –100 pA and access resistance less than 30 MΩ, which meets the criteria for most of the published data acquired with manual patch clamp. Together, we found that the automated uM Workstation successfully increased our throughput, enabling large-scale patch-clamp studies, without compromising data quality.

Using the uM Workstation we then screened top-performing single-position mutants from the field stimulation assay, including Voltron as a control. While Voltron_525_.V74G and Voltron_525_.V74W were the top performers from the field stimulation assay, their fluorescence response to a 100 mV voltage step was lower than that of Voltron2_525_ (Fig. 2b). The other mutants were also 8% to 55% less sensitive to 100 mV voltage steps than Voltron2 (Fig. S3a). Meanwhile, Voltron2_525_ was found to be 65% more sensitive than Voltron, consistent with the field stimulation assay. Furthermore, in the physiologically relevant sub-threshold voltage range (−90 to −50 mV), Voltron2_525_ exhibited an almost three-fold higher slope than Voltron_525_ (0.54±0.01 and 0.21±0.01%/mV, respectively; mean ± s.e.m., n=8 cells each, p=0.0009, Mann-Whitney U test), making it a higher-fidelity optical reporter of changes in sub-threshold membrane potential.

Surprisingly, the combo mutation screen (second round of the field stimulation assay, Fig. 1b) yielded few variants with improved sensitivities. We nevertheless screened the top 34 combo mutants using the uM Workstation. As was the case with the single-position mutants, we found no combo mutants that out-performed Voltron2_525_ (Fig. 2c, Fig. S3b). Therefore, for the remainder of this study, we focused on characterization of Voltron2_525_.

Voltron2_525_ exhibited fast onset and decay kinetics that were best fit with a double exponential (Fig. 2d). Interestingly, the A122D mutation completely eliminated the transient peak in the fluorescence response of Voltron_525_ (Fig. 2b inset). The fast and slow components of the onset and decay kinetics were similar between Voltron2_525_ and Voltron_525_ (fast onset: 0.67±0.03 ms vs 0.85±0.06 ms, fast decay: 0.89±0.09 ms vs 1.13±0.08 ms, slow onset: 3.26±0.47 ms vs 4.76±0.92 ms, slow decay: 6.27±1.41 ms vs 4.74±0.32 ms; Voltron2 and Voltron, respectively; mean ± s.e.m.; n=4 cells each). The fast component of Voltron2_525_ accounted for a significantly larger percentage of the overall response in the onset but not decay response (Fig. 2e). Overall, the kinetic properties of Voltron_525_ and Voltron2_525_ were found to be similar, consistent with the field stimulation assay.

Compared to Voltron, Voltron2 was also superior in its sensitivity to APs. Voltron2_525_ reported single APs with $\text{ΔF}/{\text{F}}_{0}$ of 10.09±1.47% (mean ± s.e.m.; Voltron_525_: n=5 cells, Voltron2_525_: n=7 cells), significantly higher than Voltron_525_ (6.16±0.74%, mean ± s.e.m, Fig. 2 f,g). The amplitudes and widths of the elicited APs were not significantly different between Voltron_525_ and Voltron2_525_ (AP amplitudes: 94.9±3.3 mV (Voltron_525_), 90.1±2.1 ms (Voltron2_525_), p=0.227, Student’s t test; AP half-widths: 1.5±0.07 ms (Voltron_525_), 1.6±0.1 ms (Voltron2_525_), p=0.813, mean ± s.e.m, Student’s t test). Both Voltron2_525_ and the soma-tagged version of the sensor (Voltron2_525_-ST) showed good membrane localization, qualitatively similar to their Voltron counterparts (Fig. S4). The baseline fluorescence of Voltron2_525_ was observed to be 30–50% lower than Voltron_525_ (Fig. 2h, Fig. S5a). The addition of a soma localization tag to Voltron2_525_ increased its sensitivity to a 100 mV depolarization pulse by ~18% (Fig. S5b). In culture, Voltron2_525_ photobleached slightly, but not significantly, slower than Voltron_525_ (Voltron_525_: 45±2%, mean ± s.d., Voltron2_525_: 41±1% reduction in fluorescence over 10 min; p=0.11, Mann-Whitney U test; Fig. 2i). Voltron2 exhibited ~−75 pA photocurrent at illumination onset, and negligible photocurrent at steady-state illumination (Fig. S5c).

We reasoned that the A122D mutation responsible for the increased sensitivity of Voltron2_525_ could have beneficial properties when grafted onto Ace rhodopsin-based GEVIs. We tested this hypothesis first in Ace2N-mNeon and VARNAM. As expected, adding the A122D mutation to both GEVIs increased their sensitivity to depolarizing and hyperpolarizing voltage pulses (Fig. S5d,e). Similar to Voltron2_525_, A122D significantly increased the slope of the sensors in the sub-threshold range (Ace2N-mNeon: 0.091±0.012 %/mV, Ace2N-mNeon.A122D: 0.303±0.012%/mV, p=0.006; VARNAM: 0.104±0.012%/mV, VARNAM.A122D: 0.147±0.010/mV, mean ± s.e.m, p=0.045; Mann-Whitney U test). The mutation eliminated the transient peak from VARNAM but not from Ace2N-mNeon. We then grafted A122D onto Positron, which did not result in increased sensitivity (Fig. S5f); however this was not surprising given that the proton transport pathway in Positron is different from Voltron^20^. Together, the results suggest that the A122D mutation appears to generalize across different FRET donors.

### Voltage imaging and stimulation in acute brain slices

**Figure 3:**
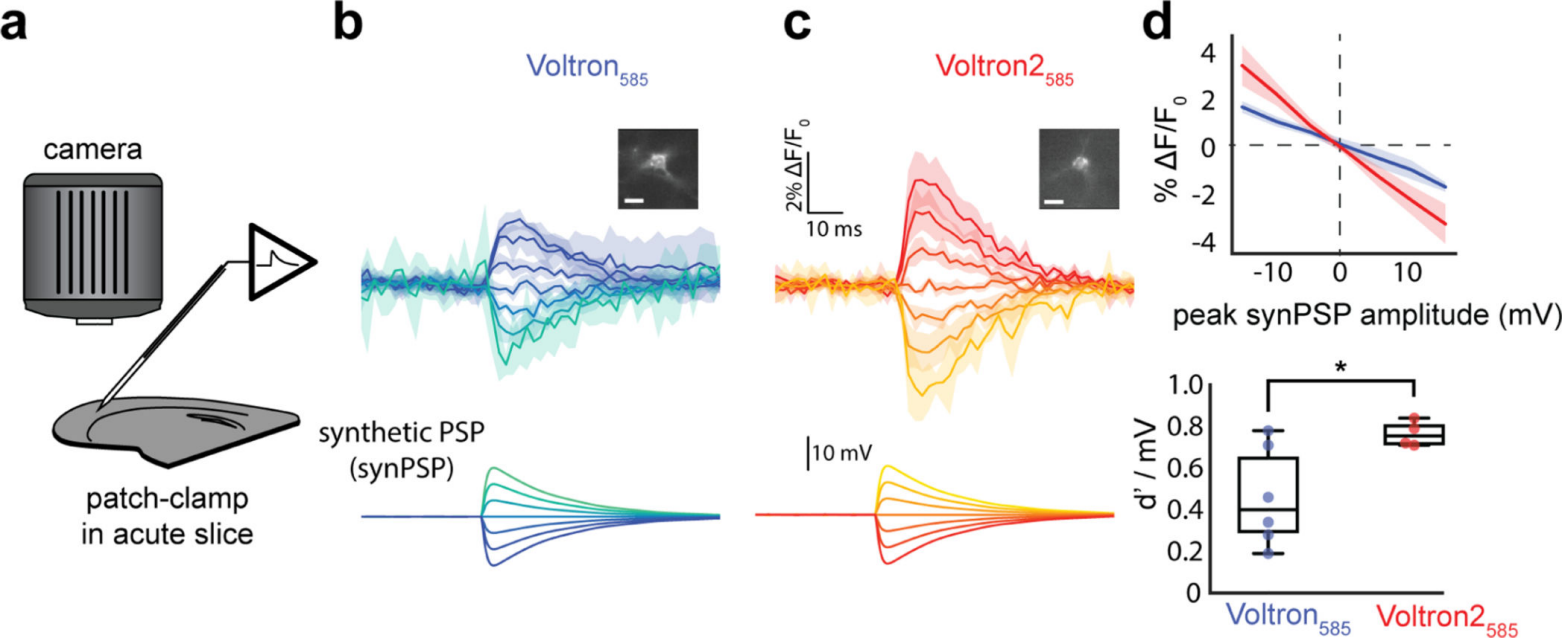
Synthetic PSP (synPSP) detection using Voltron2 in mouse brain slices. a. Experimental setup in acute mouse brain slice. b,c. Percent change in fluorescence over time for Voltron_585_ (b; n=6 cells; 2 mice) or Voltron2_585_ (c, n=4 cells; 2 mice) in response to changes from resting membrane potential of −15mV to +15mV in 5mV increments (lower panels), intended to mimic typical inhibitory or excitatory synaptic transmission. Solid lines: mean; shading: s.d.. A representative cell for each construct is shown in the inset (scale bar = 10μm). d. Top: percent change in fluorescence as a function of the peak amplitude of the synthetic postsynaptic potential (synPSP) applied to the cell (solid line: mean, shading: s.d.). Bottom: sensitivity index (d’/mV) of Voltron2_585_ is significantly higher than that of Voltron_585_ (p=0.025, Welch’s t-test). See also Fig. S6.

The high sensitivity of Voltron2 in the sub-threshold range of voltages should make it a suitable indicator for detecting low-amplitude voltage fluctuations, such as those arising as a result of synaptic activity. To test this, synthetic PSPs (synPSPs) were injected into neurons expressing Voltron_585_ and Voltron2_585_ in acute mouse brain slices (Fig. 3a). Optically captured responses to PSPs were ~40% larger for Voltron2_585_ than Voltron_585_, consistent with the improved sensitivity of Voltron2_585_ in the sub-threshold range (Fig. 3b,c; single trials: Fig. S6a-c). The overall $\text{ΔF}/{\text{F}}_{0}$ in response to ±15 mV synPSPs was ±3% for Voltron2_585_, compared to ±1.6% for Voltron_585_ (Fig. 3d top). Due to the increased sensitivity, the detectability of synPSPs was found to be significantly improved for Voltron2_585_ (Fig. 3d bottom, single cells: Fig. S6d). Together, we found that Voltron2_585_ could be used to image stimulus-locked millivolt-scale synaptic events.

**Figure 4:**
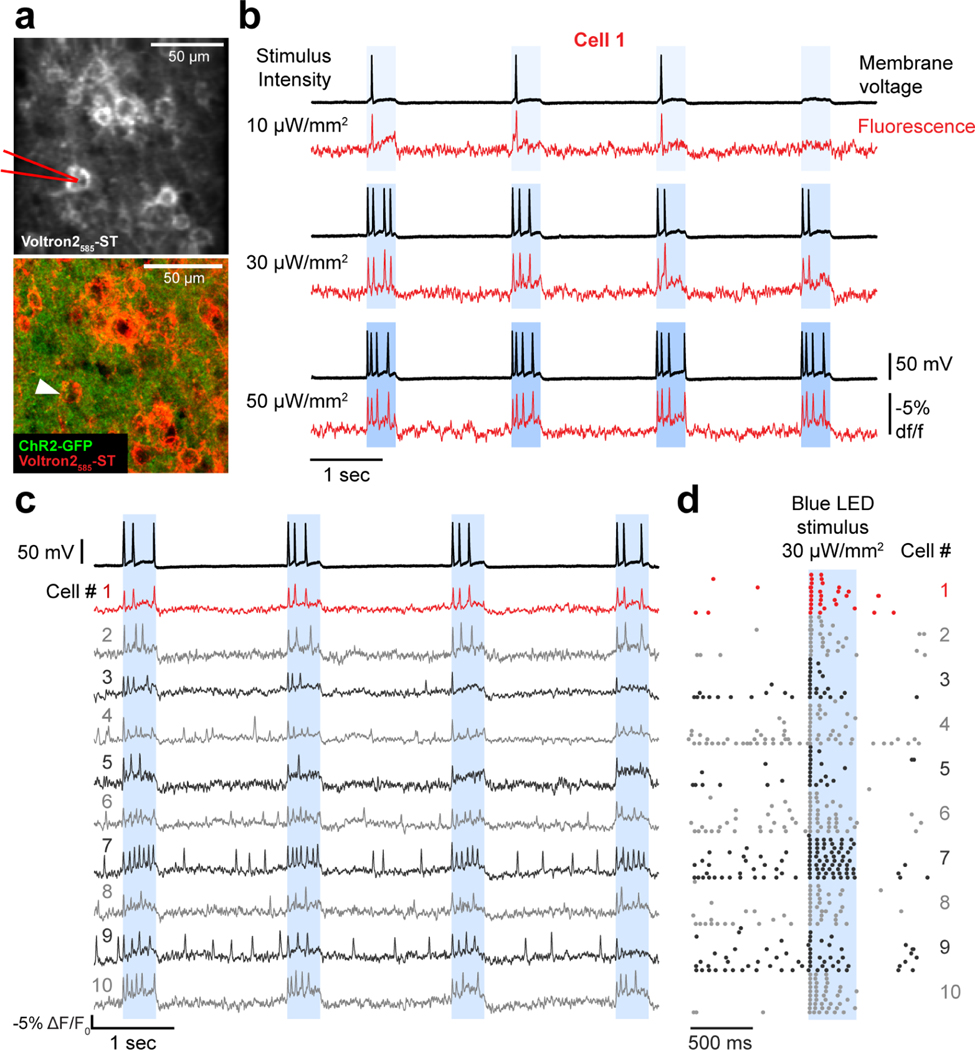
Simultaneous voltage imaging and optogenetic stimulation. a. (Top) Average intensity projection of 457 Hz confocal images showing Voltron2-expressing cells labeled with JF_585_ in an acute slice of motor cortex (n=1 mouse). Pipette used for whole-cell recordings illustrated in red. (Bottom) Post-hoc confocal image showing pan-neuronal expression of ChR2-GFP in the same field of view (FOV) shown in top panel, with patched cell #1 indicated by white arrow. b. Whole-cell membrane voltage (black traces) and corresponding Voltron2 fluorescent signal (red traces) from patched cell #1 shown in a, showing responses to 400 ms stimulation with 10 (top), 30 (middle), and 50 μW/mm^2^ (bottom) blue light. c. Voltron2_585_ signals (red and gray traces) recorded across 10 distinct cells in the FOV shown in (a) in response to 400 ms stimulation with 30 μW/mm^2^ blue light. Corresponding membrane voltage is shown for patched cell # 1 (upper black trace). d. Raster plots show trial-aligned APs detected in fluorescent signals from cells #1–10 shown in (a) and (c), across 10 repeated 400 ms blue stimulus trials. See also Fig. S7.

We then evaluated the ability of Voltron2 to be used in the context of all-optical electrophysiology. Here, we expressed Voltron2_585_-ST along with ChR2-GFP^27^ in acute slices of mouse motor cortex (Fig. 4a). We confirmed with whole-cell electrophysiology that ChR2 could reliably elicit spiking activity when illuminated with moderate blue light intensity (30 μW/mm^2^) and that Voltron2_585_ accurately tracked the membrane voltage (Fig 4b) when illuminated with yellow light. Using the same illumination intensity, we imaged a FOV with 10 neurons during repeated ChR2 activation and found robust Voltron2_585_ signals that reported expected increases in spiking activity during ChR2 stimulation (Fig. 4c,d). However, we found that the imaging light at this power is likely cross-activating ChR2. In cultured neurons electroporated with ACAGW-ChR2-Venus, yellow light (20–40 mW/mm^2^) induced large membrane depolarization (25–35% of maximum achieved by blue light) and photocurrents (5–7% of maximum achieved by blue light) (Fig. S7a-c). Concurrent yellow and blue irradiation did not produce compounded effects (Fig. 7d). Together, these results suggest that while Voltron2_585_ is nominally compatible with ChR2 for all-optical electrophysiology, using Voltron2 with a more red-shifted dye or using a more blue-shifted actuator would be necessary for reduced cross-excitation.

### *In vivo* voltage imaging of olfactory sensory neurons in zebrafish

**Figure 5:**
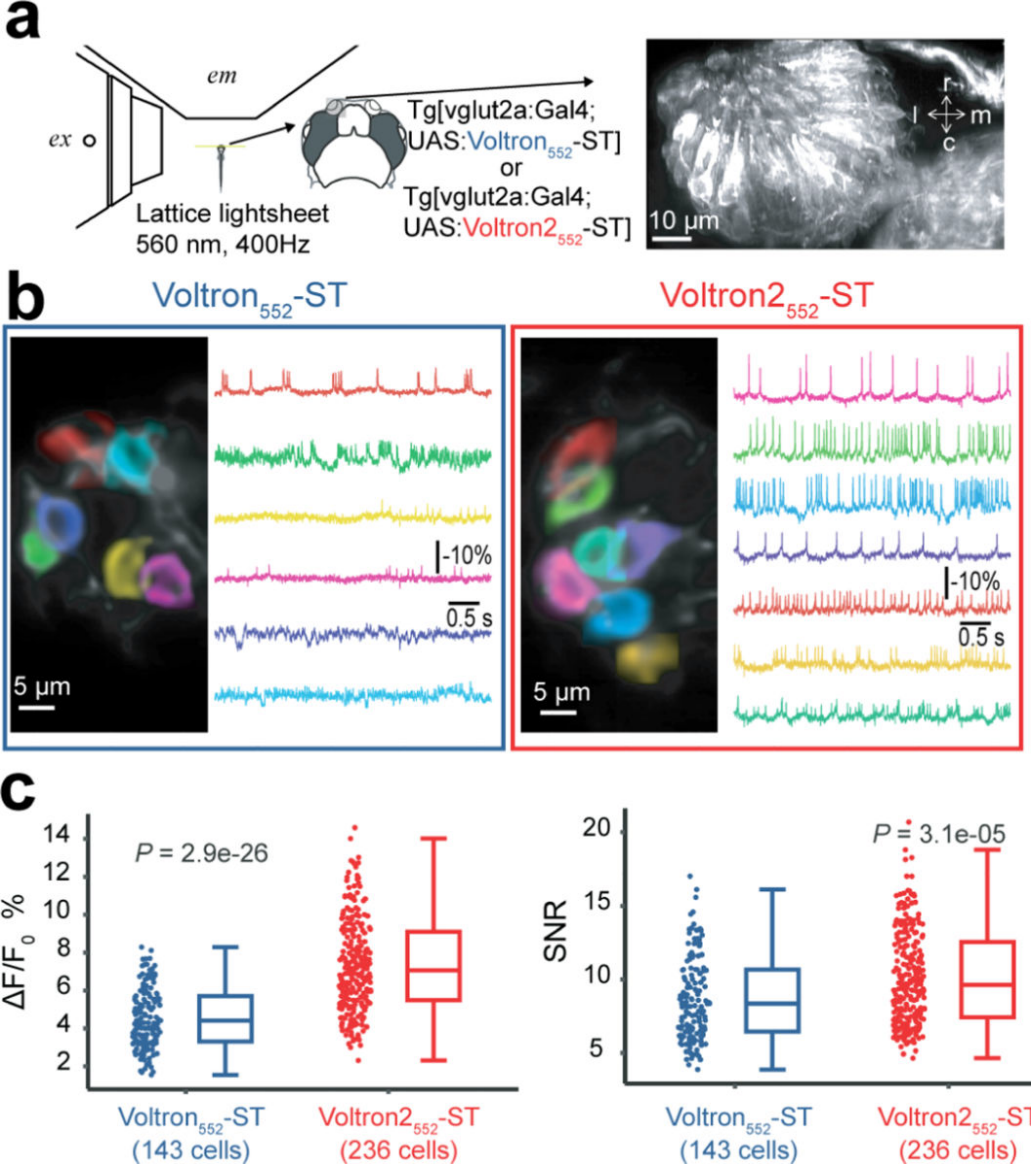
*In vivo* comparison of Voltron-ST and Voltron2-ST in zebrafish olfactory sensory neurons. a. Experimental setup. Left: Olfactory sensory neurons expressing Voltron-ST or Voltron2-ST, labeled with JF_552_ and imaged at 400 Hz using a lattice-lightsheet microscope. ex: excitation objective lens, em: imaging objective lens. Right: Volumetric rendering of olfactory sensory neurons in the nasal cavity. r, rostral; c, cadual; m, medial; l, lateral b. Representative FOVs and recordings. Spatial weights optimized for individual spiking neurons are shown in distinct colors over the structural image (left). The activity trace of corresponding neurons is shown in the same color (right). c. Performance comparisons of Voltron_552_-ST and Voltron2_552_-ST. Left: Distribution of spike-related fluorescence change of Voltron_552_-ST and Voltron2_552_-ST. Right: Distribution of SNR of Voltron_552_-ST and Voltron2_552_-ST. P values: Wilcoxon rank-sum test.

We next tested Voltron2_552_ side-by-side with Voltron in olfactory sensory neurons in larval zebrafish using a lattice lightsheet microscope (Fig. 5a). Volton2_552_ exhibited higher-amplitude spontaneous spiking and subthreshold activity than Voltron_552_ (Fig. 5b). The $\text{ΔF}/{\text{F}}_{0}$ and SNR of detected spikes was significantly higher for Voltron2_552_, measured across hundreds of cells (Fig. 5c). Both Voltron_552_ and Voltron2_552_ were imaged over 5 minutes, with voltage signals still clearly visible at the end of the experiment, suggesting that longer recording sessions are also possible.

### *In vivo* voltage imaging in adult *Drosophila melanogaster*

**Figure 6:**
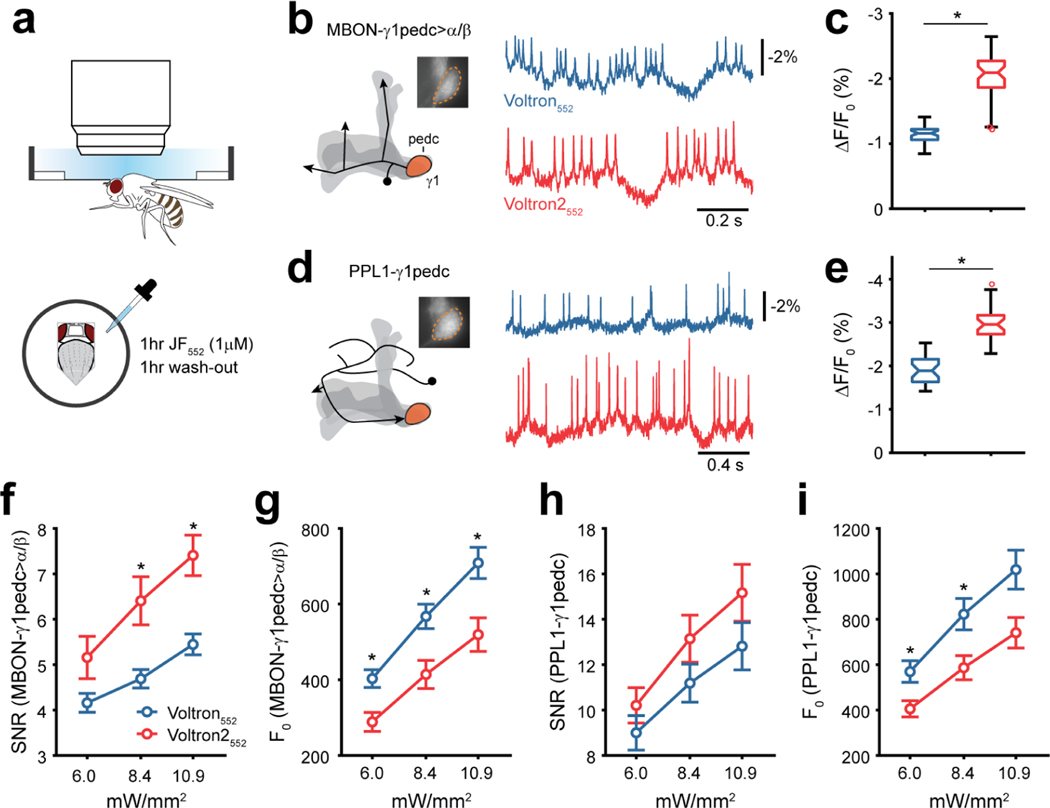
Imaging of voltage activity *in vivo* in flies. a. Experimental setup. A head-fixed fly is imaged using an sCMOS camera at 800 Hz. Voltron is loaded with JF_552_-Halotag ligand via a one-hour incubation/one-hour wash-out protocol. b,d. Voltage recordings in MBON-γ1pedc>α/β (*MB112C-Gal4*) and PPL1-γ1pedc (*MB320C-Gal4*). Neuron schematics are shown for the left hemisphere with the MB in shaded gray (arrowheads indicate axonal outputs). Fluorescence images were acquired from the γ1 compartment (inset, 50 μm × 50 μm), which contains dendrites of MBON-γ1pedc>α/β and axon terminals of PPL1-γ1pedc. Single-trial recordings of $\text{ΔF}/{\text{F}}_{0}$ traces are shown (8.4 and 6.0 mW/mm^2^ for b and d respectively). c. Spike amplitude with Voltron2_552_ and Voltron_552_ in MBON-γ1pedc>α/β. p=6.4×10^−14^, Wilcoxon rank sum test. For Voltron2_552_, the data set was from 15 hemispheres (8 flies) at three levels of illumination for a total of 45 experiments, for Voltron_552_, 13 hemispheres (7 flies) with 39 experiments. Box represents interquartile range (IQR), center represents median, notch represents 95% CI, and whiskers indicate 1.5xIQR. e. Spike amplitude in PPL1-γ1pedc. p=5.0×10^−11^, Wilcoxon rank sum test. For both Voltron2_552_ and Voltron_552_, the dataset was from 10 flies at three levels of illumination for 30 total experiments. f,h. SNR calculated as spike amplitude over standard deviation of the spike-free zones of the trace. p=0.07, 0.006, 0.003 between Voltron2_552_ and Voltron_552_ in MBON-γ1pedc>α/β, p=0.28, 0.16, 0.17 in PPL1-γ1pedc, Student’s t-test. g,i. Lower basal fluorescence levels with Voltron2_552_. p=0.0027, 0.0048, 0.0046 in MBON-γ1pedc>α/β, p< 0.05 in PPL1-γ1pedc, Student’s t-test. p=0.0132, 0.0148, 0.02. Values in (f-i) shown as mean ± s.e.m. *p<0.05, no correction for multiple comparisons was performed.

We tested Voltron2 in voltage recordings of spontaneous activity from two neuron types in the mushroom body (MB) circuit of adult *Drosophila melanogaster*, the output neuron MBON-γ1pedc>α/β and the dopaminergic neuron PPL1-γ1pedc (Aso et al. 2014) (Fig. 6a). The expression of Voltron2 was driven by split Gal4 lines (*MB112C* and *MB320C*), which uniquely target these neurons, enabling a well-matched comparison of sensor performance across different flies. We imaged both cell types in the γ1 compartment, which contains the dendritic processes of MBON-γ1pedc>α/β and the axonal terminals of PPL1-γ1pedc (Fig. 6 b,d [left]). Among several JF dyes we tried in *Drosophila* neurons, we found that JF_552_ allowed for prolonged Voltron imaging, which in PPL1-γ1pedc can last over 20 min without significant deterioration of the health of the cell. JF_552_ is a JF_549_ analogue with fluorine substitution on the xanthene ring, which shows improved cell and tissue permeability^28^. Spike amplitudes measured with Voltron2_552_ were significantly larger when compared to Voltron_552_ (Fig. 6b,d [right]). The mean spike size was 74% larger in MBON-γ1pedc>α/β (Fig. 6c), and 57% larger in PPL1-γ1pedc (Fig. 6e). The SNR of Voltron2_552_ was significantly higher in MBON-γ1pedc>α/β but not in PPL1-γ1pedc neurons (Fig. 6f,h). The basal florescence levels are lower with Voltron2 (Fig. 6g,i), which likely contributed to the more moderate improvement of SNR as compared to $\text{ΔF}/{\text{F}}_{0}$.

### *In vivo* voltage imaging in mouse hippocampus, visual cortex and motor cortex

**Figure 7:**
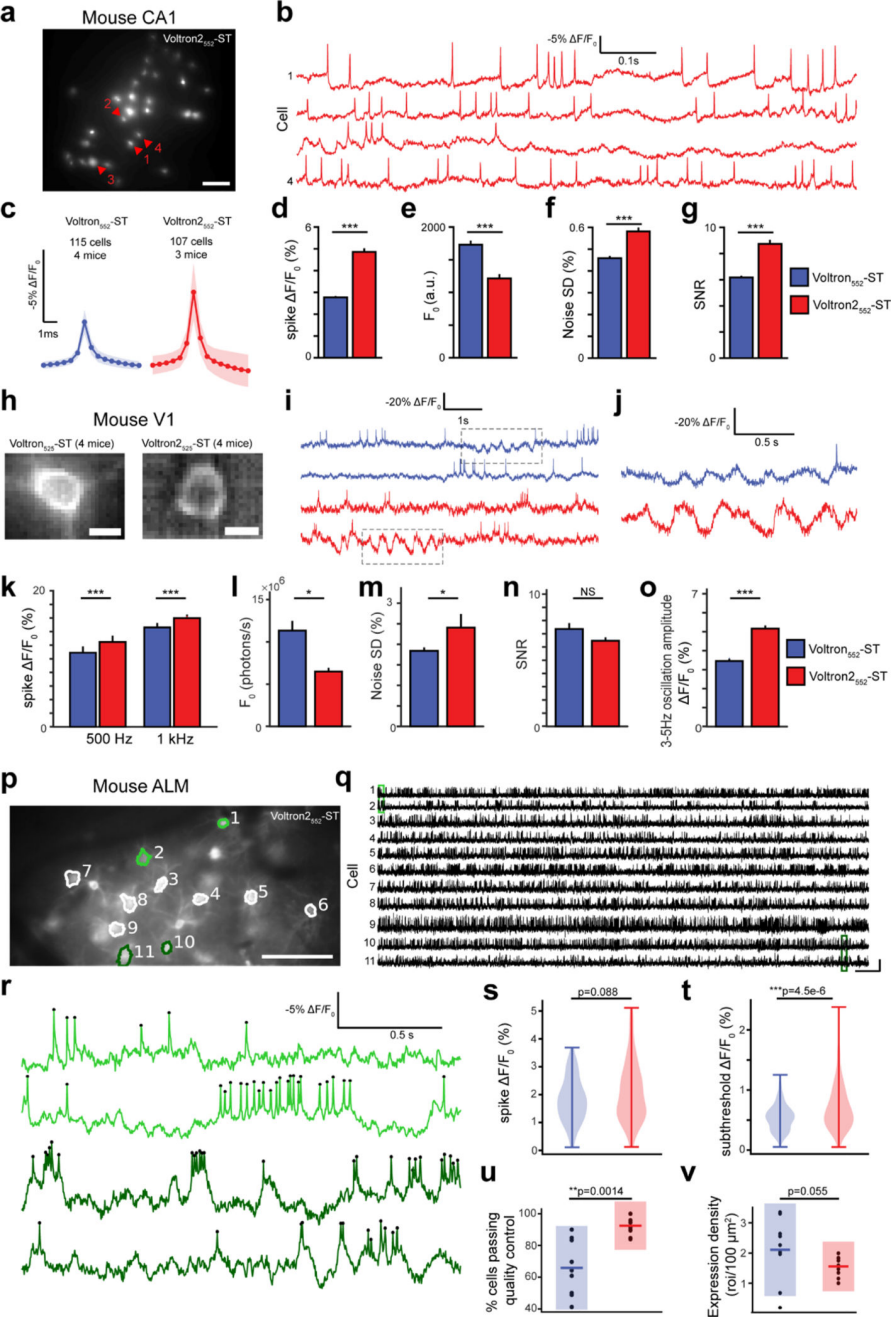
Imaging of voltage activity *in vivo* in mouse hippocampus and cortex with Voltron and Voltron2. a. Example image of hippocampal PV neurons expressing Voltron2-ST labeled with JF_552_. Scale bar: 200 μm. b. Sample fluorescence traces of cells 1–4 in (a). c. Spike waveforms of cells expressing Voltron_552_-ST or Voltron2_552_-ST. Solid line: mean; shading: s.e.m. d-g. Comparison of Voltron_552_-ST and Voltron2_552_-ST spike amplitude (d), baseline fluorescence (e), noise standard deviation (f), and SNR (g) in hippocampal PV neurons (mean ± s.e.m.). N values indicated in (c). h. Example images of cortical pyramidal neurons expressing Voltron_525_-ST (top) or Voltron2_525_-ST (bottom) labeled with JF_525_. Scale bar: 10 μm. i. Example fluorescence traces from individual neurons recorded using Voltron_525_-ST (blue) and Voltron2_525_-ST (red) detrended using a 5s median filter. Grey dashed boxes indicate detection of 3–5Hz oscillations shown in (j) and quantified in (o). j. Zoomed portions of the fluorescence traces in (i) showing spikes and 3–5Hz oscillations. k-o. Comparison of Voltron_525_-ST and Voltron2_525_-ST spike amplitude at both 500 and 1000 Hz imaging rates (k), baseline fluorescence (l), noise standard deviation (m), SNR (n), and 3–5Hz oscillation amplitude (o) in cortical pyramidal neurons (mean ± s.e.m.; 107 neurons expressing Voltron_552_-ST in 4 mice, 102 expressing Voltron2_552_-ST in 4 mice). p. NDNF interneurons in mouse ALM expressing Voltron2_552_-ST. Scale bar: 100 μm. q. $\text{ΔF}/{\text{F}}_{0}$ traces during 3 min of recording at 400 Hz from neurons shown in (p), in decreasing order of SNR. Scale bars are 10 s and −5% $\text{ΔF}/{\text{F}}_{0}$. r. $\text{ΔF}/{\text{F}}_{0}$ traces from color-coded regions of (q) with action potentials represented as black dots. s-v. Comparison of Voltron_552_-ST and Voltron2_552_-ST spike amplitude (s), subthreshold $\text{ΔF}/{\text{F}}_{0}$ (t), % cells passing quality control (u), and expression density (v) (245 neurons expressing Voltron_552_-ST in 5 mice, 181 expressing Voltron2_552_-ST in 2 mice). For all plots: Statistically significant differences between groups were determined by two-sided Wilcoxon rank-sum test. *p < 0.05, **p < 0.01, ***p < 0.001. See also Fig. S8.

We next tested Voltron2-ST *in vivo* in parvalbumin (PV) expressing interneurons in the CA1 region of the mouse hippocampus. Cells expressing Voltron2_552_-ST were illuminated using a DMD-based patterned illumination microscope, and the fluorescence responses of up to 34 neurons were imaged simultaneously at 2 kHz (Fig. 7a,b) and compared to Voltron_552_-ST (Fig. S8a,b). Spontaneous APs in PV-positive interneurons induced nearly two-fold larger fluorescence changes in Voltron2_552_ compared to Voltron_552_ expressing neurons (Fig. 7c,d). The baseline fluorescence was dimmer for Voltron2_552_ (Fig. 7e), leading to slightly larger recording noise (Fig. 7f), yet the overall SNR was still significantly improved compared to Voltron_552_ (Fig. 7g). The number of visually identifiable neurons was comparable despite the dimmer baseline fluorescence (Fig. S8c). Furthermore, photobleaching was significantly slower in Voltron2_552_ compared to Voltron_552_-expressing cells (Fig. S8d; 9.9±5.8% vs. 15.6±4.6% mean ± s.e.m in 3 minutes, respectively; p<0.001, Wilcoxon rank-sum test, n=115 Voltron_552_-expressing cells, n=105 Voltron2_552_-expressing cells).

Voltron2_525_-ST was then evaluated and benchmarked against Voltron_525_-ST in the mouse primary visual cortex. We used one-photon epifluorescence microscopy with structured illumination as previously^19^. The chronic cranial window and a mixture of Cre-dependent Voltron and dilute CaMKIIa-Cre viruses enabled sparse but bright labeling of pyramidal neurons (Fig. 7h). APs and subthreshold fluctuations were clearly observable using both sensors (Fig. 7i,j). Voltron2_552_-ST produced larger $\text{ΔF}/{\text{F}}_{0}$ responses from spikes in cortical pyramidal neurons at two acquisition speeds (Fig. 7k). Similar to other preparations, we observed that Voltron2_552_-ST was dimmer than Voltron_552_-ST (Fig. 7l). As shot noise is dominant in high-speed imaging, we observed smaller relative noise in the brighter Voltron_552_-ST-expressing neurons compared to Voltron2_552_-ST-expressing neurons (Fig. 7m). There was no significant difference in SNR between the two sensors in this preparation (Fig. 7n), likely because the improved $\text{ΔF}/{\text{F}}_{0}$ of Voltron2_552_ was offset by its higher noise. We subsequently focused on the improved sensitivity of Voltron2_552_ around resting membrane potential. Low-frequency membrane voltage oscillations in individual cortical neurons in awake mice have previously been observed in the barrel^29^, auditory^30^ and visual cortices^31^. We focused on brief (1–2 s long) periods of 3–5 Hz oscillations around a ~12 mV hyperpolarized baseline, exhibiting a peak-to-peak amplitude of ~17 mV^32^. Due to the enhanced sensitivity of Voltron2_552_ in the subthreshold range, 3–5Hz oscillations were significantly more pronounced when imaging Voltron2_552_-ST, exhibiting ~50% larger amplitude (Fig. 7j,o) and significantly higher SNR (p<0.0001; Voltron_552_: 3.8±1.9 (n=107 cells), Voltron2_552_: 4.8±2.0 (n=101 cells), mean ± s.d.; Mann Whitney U test).

We finally compared Voltron2_552_-ST with Voltron_552_-ST in NDNF-expressing interneurons in the mouse anterior lateral motor cortex (ALM) (Fig. 7 p). Using a widefield microscope, we observed APs and subthreshold fluctuations using both sensors (Fig. 7 q,r). Voltron2_552_-ST produced significantly higher-amplitude responses to subthreshold fluctuations, but not spikes (Fig. 7 s,t). The percentage of neurons in a field of view that passed quality control was significantly higher for Voltron2_552_-ST (Fig. 7u), although the expression density was slightly, but not significantly higher for Voltron_552_-ST (Fig. 7v).

Together, these data indicate that Voltron2_552_ significantly improves the quality of *in vivo* voltage imaging in multiple regions of the mouse brain.

### Imaging the subthreshold basis of spike synchrony between hippocampal PV cells

**Figure 8:**
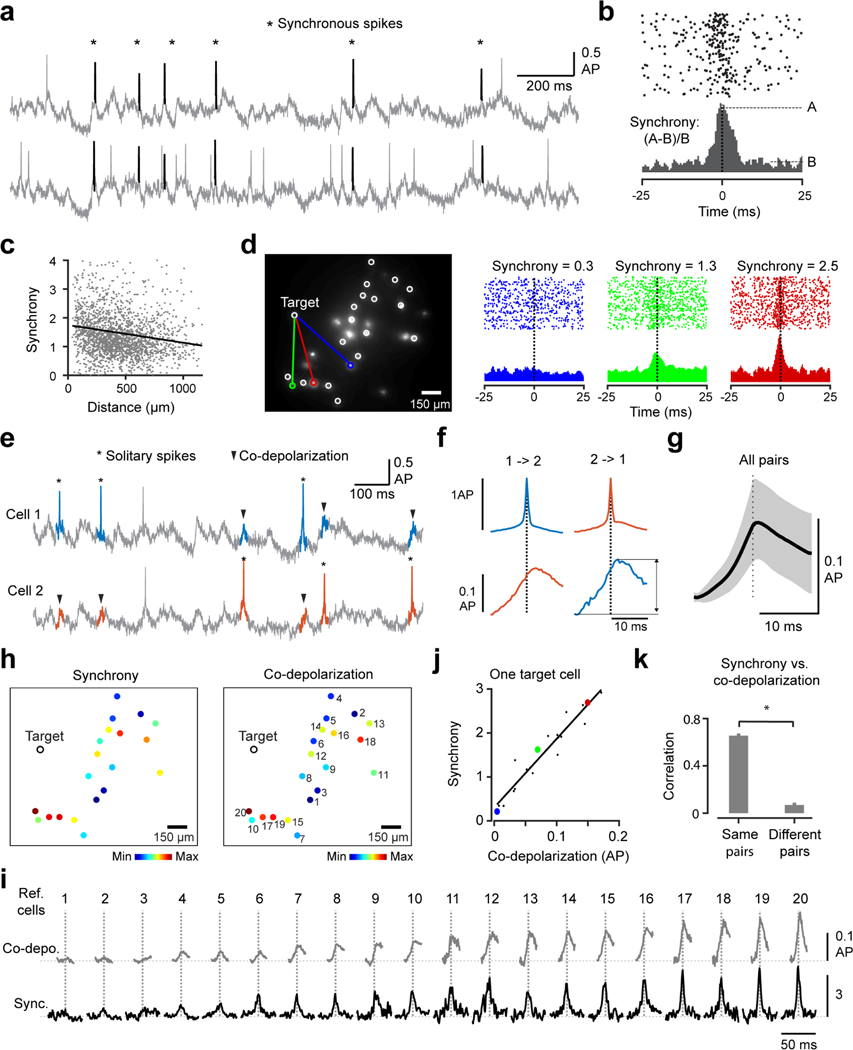
Co-depolarization of hippocampal PV interneurons in mice. a. Example fluorescence traces of a pair of PV neurons showing their synchronous action potentials (asterisks). b. Spike cross-correlogram (CCG) of the same pair of neurons in (a). c. Synchrony strength plotted against the distance between cells (n=4,376 pairs, 7 mice). d. Spatial locations (left) and CCGs (right) of three representative PV cell pairs showing varying strength of spike synchrony. e. Example fluorescence traces (the same cells as in a) showing solitary spikes (asterisks) and co-depolarization in the other cell (arrowheads). f. Averaged spike (top) and co-depolarization (bottom) of the two neurons in (e). The double arrow indicates the size of co-depolarization. g. Co-depolarization averaged over all cell pairs. The dashed vertical line indicates the time of the reference spikes. Solid line: mean, shading: s.d. h. Maps of spike synchrony (left) and co-depolarization (right) of a target neuron (shown by a white circle) relative to each of the reference cells. Strength of synchrony and size of co-depolarization was color coded (from blue to red, minimum to maximum) and shown on each reference cell. i. Co-depolarization (top) and spike synchrony (bottom) of the target cell in (h) relative to each of the reference cells in (h). j. Correlation between synchrony and co-depolarization for the target cell shown in (h). Each dot indicates a different reference neuron. Colors correspond to the representative pairs in (d) and (h). k. Correlation between synchrony and co-depolarization measured relative to the same or different reference neurons. For the “Different pairs” condition, synchrony to a given reference neuron was correlated with the co-depolarization triggered by a different reference neuron, located at a similar distance from the target. A correlation value was computed for each target cell (n = 204) that had more than 10 simultaneously imaged reference neurons. The bars show the mean correlation values of all target cells (n=204). Error bars indicate s.e.m. *p<10^−47^, Student’s paired t test.

Finally, we leveraged the improved SNR and subthreshold sensitivity of Voltron2 to study the basis of spike synchrony between PV cells *in vivo*. Previous studies have reported synchronous activity between inhibitory interneurons both *in vitro* and *in vivo*^33–38^. Consistent with these reports, we often observe near-synchronous APs in pairs of simultaneously imaged PV cells in the hippocampus (Fig. 8a). On average, 24 ± 15% of the spikes in PV cell pairs occurred within 5 ms of each other, compared to 5.5 ± 4.8 % when the spike times were randomly shuffled (mean ± s.d., n= 4,376 pairs). The abundance of these synchronous spikes produced a sharp peak in the spike cross-correlograms (CCG) (Fig. 8b), with a full width at half-maximum of 4.0 ± 1.2 ms. We quantified the strength of synchrony between each pair of neurons by comparing the mean spike probability in a synchronous period (|Δt|≤1.5ms) to a nearby control period (15ms≤Δt≤25ms) (Fig. 8b). The strength of synchrony decreased slightly with distance (Fig. 8c). But even for cells separated by comparable distances, the strength of synchrony varied considerably from cell to cell (Fig. 8d).

Several synaptic mechanisms have been proposed to synchronize the activity of PV neurons^34,35,37–40^. Specifically, PV cells are coupled to each other by gap junctions, which conduct APs into fast ‘spikelet’ signals in coupled cells to drive synchronous spiking^35,40^. In addition, PV cells are also known to inhibit each other through chemical inhibitory synapses^37,39^ and share common excitatory input from upstream pyramidal cells^34^. Such reciprocal inhibition and shared excitation have also been shown to drive the synchrony between cells^34,37,39^. To test the relative contribution of these mechanisms *in vivo*, we analyzed the subthreshold voltage of each PV cell (‘target’) near the spikes of other simultaneously imaged PV neurons (‘reference’). We focused on periods of the recordings where the target cell did not spike (within 10 ms of the reference spikes) to minimize the effect of membrane non-linearity near action potentials. Although PV cells are known to inhibit each other^39^, we found a clear net depolarization of target PV neurons near the spikes of reference PV cells, even when the target cells themselves did not spike (Fig. 8e-g). This ‘co-depolarization’ started earlier and rose several times slower than the reference spikes (Fig. 8f-g), indicating that it cannot be explained by optical crosstalk of the reference APs. The relatively slow and early depolarization is also inconsistent with a dominant spikelet contribution, in which faster, spike-like depolarization starting simultaneously or after the reference spikes is expected^35,40^. Rather, our data is consistent with the idea that the same excitatory inputs driving the reference spikes also arrive in the target cell, producing either subthreshold co-depolarization (when the target cell did not spike) or synchronous spiking (when both neurons spiked).

If subthreshold co-depolarization indeed contributes to synchronous spiking, one would expect stronger co-depolarization in cells that are more synchronized. To test this, we analyzed the correlation between the size of co-depolarization and the strength of synchrony between cells. We focused on 204 target cells that had enough simultaneously imaged reference neurons (>10) to build a meaningful correlation. For each target cell, we computed the correlation between its synchrony and co-depolarization relative to each reference cell (Fig. 8h-k). This analysis revealed a strong correlation between synchrony and co-depolarization, with the same target cell showing larger co-depolarization near spikes of more synchronized reference neurons (Fig. 8h-k). Similar to spike synchrony, co-depolarization also tended to decrease with distance. To confirm that the correlation between synchrony and co-depolarization was cell pair specific and not just due to their shared dependency on distance, we performed a control analysis. We used different reference cells, located at similar distances from the target neuron, to compute synchrony and co-depolarization, respectively. The synchrony and co-depolarization were no longer correlated when they were measured relative to different reference neurons (Fig. 8k, ‘different’ pairs), indicating that their correlation was cell-pair specific. Together, these data support a role of subthreshold co-depolarization, possibly reflecting shared excitatory input^34^, in driving synchronous spiking of PV cells in vivo.

## Discussion

Here we present the voltage indicator Voltron2 and demonstrate its performance in cell culture, brain slices, and *in vivo*. Compared to Voltron, Voltron2 is more sensitive to APs and is approximately 3-fold more sensitive to subthreshold changes due to its steeper slope around the resting membrane potential. The photostability of Voltron2 is equivalent to or slightly better than Voltron across preparations, suggesting that it would also compare favorably with other GEVIs^19^. Voltron2-ST is ~2.5-fold more sensitive than Positron-ST in cultured neurons. As with our efforts to engineer positive-going FRET sensors^20^, the mutation we discovered generalized to Ace-based GEVIs with fluorescent protein reporters. Perhaps the sensitivity-improving mutations identified in our screen will also be useful for optimization of rhodopsin-only GEVIs, such as those based on Arch, that rely on imaging the dim retinal fluorescence directly.

Engineering improved voltage indicators has been more challenging than GECIs. In this study, we screened >2,700 variants to attain ~50% increase in $\text{ΔF}/{\text{F}}_{0}$ in the Voltron scaffold. Applying the same mutagenesis and screening strategy to GCaMP, RCaMP and R-GECO1 calcium indicators yielded >500% increases in $\text{ΔF}/{\text{F}}_{0}$ with fewer than 1,000 screened variants^41,42^. Further, combining mutations in GCaMP scaffolds has often yielded additive benefits, while doing so in the current context of the Ace2N rhodopsin ultimately did not produce any variants with significant improvements over the best single A122D mutation. The reason for this is not apparent. It is possible that there are biophysical phenomena that impose a ceiling on the sensitivity of this scaffold. For example, it is expected that the FRET efficiency between the HaloTag dye or FP donor and the rhodopsin retinal acceptor will limit the maximum fluorescence change. Each of these chromophores resides on or in a bulky protein domain, limiting their closest approach distance. Further, it is not clear why the A122D mutation alone improves the sensitivity of Voltron2. Mutations at the equivalent position in Bacteriorhodopsin (position A126 in Bacteriorhodopsin numbering) changed the thermal stability of that protein^24^; however, other stability-altering mutations that we tested did not yield successful sensors. We were intrigued to observe that A122D decreased the sensor’s fluorescence at the resting membrane potential. It seems possible that additional mutations could restore the original resting fluorescence of Voltron while maintaining the improved sensitivity of A122D, leading to improved SNR, but our screens failed to identify such a variant.

Various high-throughput platforms have been developed that have been used to screen for improved GEVIs^11,14,43,44^. The majority of these platforms utilize bacteria or tissue culture cells for screening. We instead opted to perform our high-content primary screen in dissociated neurons, a costlier and more time-consuming strategy, but one that maximized the compatibility of the resulting sensor with *in vivo* neuronal imaging. Still, our field stimulation assay was insufficiently sensitive to disambiguate the top-performing sensors. This was mainly due to well-to-well as well as week-to-week variability of the responses. We therefore relied on the automated patch-clamp system that afforded us the ability to screen dozens of sensors faster than possible manually, without compromising data quality. The system had a lower throughput than the field stimulation assay but enabled us to characterize the sensitivity and kinetics of many variants with much higher fidelity. The combination of both field stimulation and patch-clamp screens provided a high-quality assessment of top-performing variants.

We show that like its predecessor, Voltron2 can be readily used for *in vitro* and *in vivo* imaging in mice, flies, and fish. In brain slices, we performed proof-of-principle experiments to demonstrate the ability to detect stimulus-locked subthreshold activity, which could be useful for electrode-assisted or all-optical connectivity mapping^45–49^. *In vivo*, we show significant improvements in the detection of 10–20 mV membrane voltage oscillations. The improvements we saw between Voltron and Voltron2 were generally consistent among the dye ligands that were tested *in vivo* (JF_525_, JF_552_, and JF_585_).

Given the richness of information contained within subthreshold activity *in vivo*, including excitatory and inhibitory PSPs, oscillations of various frequencies, spikelets, and other features, a voltage indicator with high single-cell sensitivity in the subthreshold range would be highly impactful. Notably, recent engineering efforts on the ASAP ^50–52^, Ace-mNeon2 ^15^ and Arch ^7,53–55^ scaffolds have made great strides towards this capability. Here, we also demonstrated the ability of Voltron2 to capture subthreshold activity, and applied it to study the phenomenon of AP synchronization in hippocampal PV interneurons in mice. The synchronous subthreshold depolarization patterns of neighboring cells suggested the presence of shared excitatory inputs in the network. However, the possible role of gap junctions in synchronizing slow depolarization between interneurons cannot be excluded^56^.

Increasing the sensitivity of voltage indicators (the difference in photon flux per millivolt change in membrane potential) and reducing photobleaching still remain the main challenges to increase their adoption for *in vivo* experimentation. For all-optical electrophysiology experiments, more developments are still needed to minimize optical cross-excitation when using voltage indicators and optogenetic actuators in the same preparation. Chemigenetic indicators offer the exciting ability to use finely-tuned, narrow-spectrum dyes which could help in maintaining orthogonal excitation spectra of actuators and sensors. Protein engineering efforts devoted to creating two-photon-compatible indicators will also be required to address need to image deep in the brain while maintaining single-cell resolution. Chemigenetic indicators like Voltron2 continue to be promising scaffolds to address these goals.

## STAR Methods

### Resource Availability

#### Lead contact

Further information and requests for resources and reagents should be directed to and will be fulfilled by the lead contact, Ilya Kolb (kolbi@janelia.hhmi.org).

#### Materials availability

The following plasmids and associated DNA sequences used in this study are available on Addgene:

- pAAV-syn-FLEX-Ace2N-4AA-mNeon-ST A122D WPRE (#172908)
- pGP-pcDNA3.1 Puro-CAG-Voltron2 (#172909)
- pGP-pcDNA3.1 Puro-CAG-Voltron2-ST (#172910)
- pGP-CAG-Ace2N-4AA-mNeon A122D-WPRE-bGH-polyA (#172911)
- pGP-CAG-Ace2N-4AA-mNeon-ST A122D-WPRE-bGH-polyA (#172912)
- pGP-CAG-VARNAM A122D WPRE-bGH-polyA (#180486)

The JF_549_-HaloTag ligand is available from Promega; all other dyes can be requested at dyes.janelia.org.

#### Data and code availability

- All data and analysis software are available upon request.
- Any additional information required to reanalyze the data reported in this work paper is available from the Lead Contact upon request

### Experimental Model and Subject Details

#### Neuronal cell culture

Experiments were conducted in accordance with guidelines for animal research approved by the Janelia Research Campus Institutional Animal Care and Use Committee. For neuronal cell culture, neonatal (P0) rat pups were harvested from time-pregnant rats obtained from Charles River.

#### Zebrafish

Experiments were conducted in accordance with guidelines for animal research approved by the Janelia Research Campus Institutional Animal Care and Use Committee. The following stable transgenic lines were generated for this study: Voltron (Tg[vglut2a:Gal4; UAS:Voltron-ST]) and Voltron2 (Tg[vglut2a:Gal4;UAS:Voltron2-ST]). Zebrafish were used 4–6 days post-fertilization (dpf).

#### Flies

Experiments were conducted in accordance with guidelines for animal research approved by the Janelia Research Campus Institutional Animal Care and Use Committee. The following transgenic lines were generated for this study: UAS-IVS-syn21-Ace2NHalo-p10 Su(Hw)attP8 (Voltron) or UAS-IVS-syn21-Ace2N(A122D)Halo-p10 Su(Hw)attP8 (Voltron2).

#### Mice

For *in vivo* mouse ALM imaging, experiments were conducted in accordance with guidelines for animal research approved by the Janelia Research Campus Institutional Animal Care and Use Committee. For imaging in brain slices, experiments were conducted in accordance with the guidelines for animal research approved by the Allen Institute and Boston University Institutional Animal Care and Use Committees. For *in vivo* mouse visual cortex imaging, experiments were approved by the Institutional Animal Care and Use Committee at the Second Faculty of Medicine, Charles University in Prague. For *in vivo* mouse hippocampus imaging, experiments were approved by the Institutional Animal Care and Use Committee at the National Yang Ming Chiao Tung University.

Mice used for this study were either wild-type (C57BL/6, Charles River), PV-Cre (B6;129P2-Pvalbtm1(cre)Arbr/J), or NDNF-Cre (B6.Cg-Ndnftm1.1(folA/cre)Hze/J) (Jackson Laboratory). Male and female mice were used interchangeably. Mice were housed in 12h light:dark reverse-cycle rooms.

### Method details

#### Single-site directed mutagenesis

The cloning vector pcDNA3.1/Puro-CAG- Ace2N_HaloTag expression vector (Invitrogen) was modified by moving the KpnI site from outside of the insert to the junction between Ace2N domain and the HaloTag. The subsequent vector was digested by NheI/KpnI cleaving out the Ace2N domain. End PCR primers were designed 30bp upstream of NheI site (5’-GCTCACAAATACCACT-3’) and 38 bp downstream of new KpnI site (5’-CCAGGACTTCCACATAA-3’). Overlapping internal primers were designed for each of 40 targeted amino acid residues in the Ace2N domain. One primer of the pair contained the degenerate codon NNS and the other primer contained a 27–30 bp complementary overhang. When paired with the end primers two amplicons were created (Phusion High-Fidelity DNA Polymerase; NEB) that overlap with each other and the digested vector ends. Each set of overlapping paired amplicons (37.5 fmol each) were assembled with the digested pcDNA3.1/Puro-CAG backbone (25 fmol) using an isothermal assembly reaction ^57^. Each 20 uL reaction mix consisted of 5X isothermal assembly buffer (25% PEG-8000, 500 mM Tris-HCl pH 7.5, 50mM MgCl2, 50mM DTT, 1mM each dNTP and 5mM NAD), T5 exonuclease (0.08 U, NEB), Taq DNA Ligase (80 U, NEB), Phusion HF DNA Polymerase (0.5 U, NEB). The reactions were incubated at 50°C for 30–60 min. Reactions were transformed into STABL2 chemically competent E. coli cells (ThermoFisher) and plated on LB/Amp agar plates and incubated at 37°C for 16–20 hours.

For each site library, 96 colonies were picked into 2.6 mL of 2x-YT media (2 × 1.3 mL in 2 mL deep-well culture plates) and grown for 24 hours, rotating at 225 rpm at 37°C with Ampicillin (100mg/L). The cultures were pelleted at 3200g and frozen at −80°C. For each plate plasmids were extracted using the E-Z 96 FastFilter Kit (Omega BioTek) and eluted into a half-area UV transparent 96 well plate (Corning Costar). Each of the plasmid plates was concentration normalized to 60 ng/μL by reading the 260 nm absorbance (Tecan Infinite M1000Pro) followed by custom dilution (Hamilton Nimbus). Variant plasmids were arrayed along with controls for high-throughput electroporation of neuronal cell culture (Hamilton STAR). Top-performing variants from the subsequent field stimulation assay were Sanger-sequenced to determine their mutation; the entire library was also sequenced using a next-generation deep-sequencing approach^58^.

#### Combinatorial mutagenesis

Top-performing single-site mutations (Y63L, N69E, V74E/W, R78H, N81S, L89A/C/G/T, A122D/H, V196P) were recombined to test all possible combinations (n=1,423). All combinations could be recapitulated using two overlapping amplicons covering the Ace2N domain. Some mutations (Y63L, A122D/H, V196P) were introduced as part of the PCR template and others (N69E, V74E/W, R78H, N81S, L89A/C/G/T) by PCR primer. For the N-term amplicon (305bp) twenty-four reverse primers were designed based on the wild-type anti-sense sequence (5’-AGTGGTGTGGTCAGCACCCAGTTAATATATCTTGCGTAGACCACCTGCCTTTCACCATTCATTGTCAGGTCC-3’) and included every combination of N69E, V74E/W, R78H, N81S. Forty-eight unique N-term amplicons were created by combining these twenty-four reverse mutagenic primers, the upstream end primer (5’GCTCACAAATACCACT-3’) and templates with and without Y63L. For the C-term amplicon (493bp) 10 forward primers were designed based on the wild-type sense sequence (5’- ATATTAACTGGGTGCTGACCACACCACTGCTCCTGCTCGATCTCATCGTCATGACCAAGATGGGCGGAGTGA −3’) and included every combination of N81S, L89A/C/G/T. Sixty unique C-term amplicons were created by combining 10 forward mutagenic primers, the downstream end primer (5’- CCAGGACTTCCACATAA-3’) and templates each containing a combination of A122D/H and V196P. The N-term and C-term amplicon libraries overlapped by 28 bp (5’-ATATTAACTGGGTGCTGACCACACCACT-3’) and included the N81 site in both. The PCR products were gel extracted, quantified and normalized to 18.75 fmol/μL. The NheI/KpnI digested pcDNA3.1/Puro-CAG backbone was normalized to 12.5 fmol/μL. Using a liquid-handling robot (Hamilton STAR) the N-term and C-term amplicon sets were pairwise combined (2μL each) along with the NheI/KpnI digested pcDNA3.1/Puro-CAG vector (2uL) to create 1,423 unique isothermal assembly reactions in 96 well thermocycler plates. The plates were reacted and transformed as above in 96-well plates. Approximately 35 μL of each transformant was robotically dispensed into the corresponding wells of two 48 well Q-trays (Genetix) containing LB/Amp agar. Q-trays were incubated for 16–20 hours at 37°C and two colonies were picked from each well and separately cultured, pelleted and frozen in 96 well deep well plates. Plasmids were extracted from the 96 well pellets and concentration normalized as above. Once verified by Sanger sequencing the combinatorial variants were arrayed for electroporation of neuronal cell culture and the subsequent field stimulation assay.

#### Neuronal cell culture

Neonatal rat pups (Charles River Laboratory) were euthanized and neocortices (for field stimulation experiments) or hippocampi (for patch-clamp experiments), were isolated. Tissue was dissociated using papain (Worthington) in 10 mM HEPES pH 7.4 in Hanks’ Balanced Salt Solution for 30 min at 37°C. Suspensions were triturated with a Pasteur pipette and passed through a 40-μm strainer. Cells were transfected by combining 5×10^5^ viable cells with 400 ng plasmid DNA and nucleofection solution in a 25 μL electroporation cuvette (Lonza). Cells were electroporated according to the manufacturer’s protocol.

For the field stimulation assay, neurons were plated onto poly-D-lysine (PDL) coated, 96-well, glass bottom (#1.5 cover glass) plates (MatTek) at ~1×10^5^ cells per well in 100 μL of a 4:1 mixture of NbActiv4 (BrainBits) and plating medium (28 mM glucose, 2.4 mM NaHCO_3_, 100 μg/mL transferrin, 25 μg/mL insulin, 2 mM L-glutamine, 100 U/mL penicillin, 10 μg/mL streptomycin, 10% FBS in MEM). The next day, 190 μL of NbActiv4 medium was added to each well. Plates were incubated at 37˚C and 5% CO2, to be imaged after 12–15 days in culture. Typically, 8 wells of a 96-well plate were electroporated with Voltron (as a control) and the remaining wells were electroporated with constructs of interest (4 wells per construct). The first and last columns of the plate were not used to avoid edge effects.

For patch-clamp, ~2×10^5^ cells were plated onto PDL-coated, 35-mm glass bottom dishes (Mattek, #0 cover glass) in 120 μL of a 1:1 mixture of NbActiv4 and plating medium in the center of the plate. The next day, 2 mL of NbActiv4 medium was added to each plate. Dishes were incubated for 7–13 days prior to beginning experiments.

#### Field stimulation assay in neuronal culture

To prepare the neurons for field stimulation, they were first incubated for one hour in NbActiv4 media supplemented with 2 nM JF_525_-HaloTag at 37°C. They were then rinsed three times with imaging buffer containing (in mM) 140 NaCl, 0.2 KCl, 10 HEPES, 30 glucose (pH 7.3–7.4) and left in a solution containing imaging buffer with added receptor blockers (10 μM CNQX, 10 μM (R)-CPP, 10 μM gabazine, 1 mM (S)-MCPG, Tocris) to reduce spontaneous activity ^59^.

The field stimulation assay for voltage indicators was adapted from our existing screening pipeline ^41,42^ based on an inverted microscope (IX-81, Olympus). Cells were illuminated with a white LED (Cairn Research) through a custom filter cube (excitation: 512/25 nm, emission: 555/20 nm, dichroic: 525 nm, Chroma) and imaged using a 40x/0.6 NA objective (Olympus) with an EMCCD camera (Ixon Ultra DU897, Andor). To enable high-speed imaging, an Optomask (Cairn Research) was used to mask out camera pixels outside a 256×256 center square. Reference images of each field of view (FOV) were taken at full sensor frame, 100 ms exposure. For high-speed imaging during stimulation, we applied 8x binning and 25 EM gain for a resulting frame rate of 1,497 Hz.

For each well in the 96-well plate, either 9 FOVs surrounding the center of the well were chosen, or a machine vision function utilizing ilastik (RRID:SCR_015246)^60^ was used to automatically focus on cell somata. For each FOV, first, a reference image was acquired, and then, neuronal APs were evoked by field stimulation via a custom-machined platinum electrode (8 pulses, 40 V, 1 ms, 8.3 Hz; S-48, Grass Instruments) concurrently with high-speed imaging. The camera ‘fire’ signal and the stimulator sense line were used to determine the frame at which the stimulation occurred.

To correct for photobleaching, a single decaying exponential function $p_{1}e^{-\frac{t}{p^{2}}}$ with two free parameters ($p_{1}$, $p_{2}$) was fit to the time series trace for each pixel and subtracted. Frames succeeding each electrical stimulus (during which the response nominally occurred) were excluded from the fit. The value of the fitted bleach function at the first frame was taken as the baseline fluorescence for that pixel. Background fluorescence was computed as the 1^st^ percentile of the baseline fluorescence across all pixels.

Responses to the eight electrical pulses within each recording were averaged using the timings derived from the camera and electrode triggers. For each pixel, a Mann-Whitney U test was performed between the frames preceding the average response (20 ms) and 10, 20, and 40 ms of frames succeeding it. Pixels with a p value < 0.001 for any of these three tests were considered responsive and averaged together to contribute to the $\text{ΔF}/{\text{F}}_{0}$ trace. Traces were fit with the product of a rising and decaying exponential to capture both the on and off kinetics. The fit was used to calculate the characteristics of the variant such as maximum $\text{ΔF}/{\text{F}}_{0}$ and kinetics ($\tau_{ON}$, $\tau_{OFF}$).

Pixel statistics were pooled across all the wells in each plate that contained the construct of interest. Wells with fewer than four responsive pixels were considered to be unresponsive and discarded from analysis.

As a quality control technique for every assay plate, a percent detectable improvement (PDI) statistic was calculated to answer the question: “Given the variability of Voltron control wells in the plate, what is the minimum improvement in $\text{ΔF}/{\text{F}}_{0}$ that can be reliably detected?”. That is, a PDI of 20% for a plate indicates that a ≥20% improvement in $\text{ΔF}/{\text{F}}_{0}$ over Voltron can be considered statistically meaningful. Large PDI values are undesirable because they indicate high variability in the control responses. PDI is calculated as follows: 100*(mean(x)-quantile(x,0.01))/mean(x), where x is the $\text{ΔF}/{\text{F}}_{0}$ of Voltron control well pixels, sampled 10,000 times with replacement.

Normalization to in-plate Voltron controls was useful to reduce the effects of plate-to-plate and week-to-week variability. Pixels from each variant were pooled across wells. For each variant, the median was taken from this pool and divided by the median from the control pool to perform the normalization. Significance values for each variant were determined using a Mann-Whitney U test between the pools.

#### Automated whole-cell electrophysiology

Cultured neurons were patch-clamped at 7–13 DIV at room temperature (23°C). On the day of the experiment, cell culture medium was first rinsed with imaging buffer consisting of (in mM): 145 NaCl, 2.5 KCl, 10 D-Glucose, 10 HEPES, 2 CaCl_2_, 1 MgCl_2_ (pH 7.3, adjusted to 310 mOsm with sucrose). The neurons were then incubated with 100 nM JF_525_ dye for 10 minutes (for Voltron mutant screening only), rinsed twice, and kept in imaging buffer. For voltage clamp recordings, 1 μM TTX was added to the bath to suppress the generation of APs. Micropipettes were pulled on a horizontal puller (P-97, Sutter Instruments) to a tip resistance of 3 to 6 MΩ. For voltage clamp experiments, pipettes were filled with cesium-based internal solution containing (in mM): 115 CsMeSO_4_, 15 CsCl, 3.5 Mg-ATP, 5 NaF, 10 EGTA, 10 HEPES, 3 QX-314 (pH 7.3–7.4, 280–290 mOsm). For current clamp experiments, pipettes were filled with 130 KMeSO_4_,10 HEPES, 5 NaCl, 1 MgCl_2_, 1 Mg-ATP, 0.4 Na-GTP, 14 Tris-phosphocreatine (pH 7.3–7.4, 280–290 mOsm).

To perform automated patch-clamp screening of the top-performing hits from the field stimulation assay, we used a custom-built Automated uM Workstation, manufactured by Sensapex (Oulu, Finland), based on the PatcherBot^25^. The system is built around an AxioObserver 7 inverted microscope (Zeiss), outfitted with a computer-controlled stage, micromanipulators, and pipette pressure controllers. Pipettes were automatically cleaned between every patch-clamp attempt with Tergazyme and reused, enabling higher throughput than possible with manual patch-clamp^25,61^. Electrophysiology recordings were performed with a Multiclamp 700B amplifier (Molecular Devices), and digitized with a multifunction data acquisition board (National Instruments PCIe-6259). Neurons were imaged using a 40X/1.3 NA oil immersion objective (Zeiss), illuminated with high-power LEDs (Spectra-X light engine, Lumencor) and imaged with a digital sCMOS camera (Hamamatsu Orca Flash 4.0) at 985 Hz. To image Voltron_525_, we used a filter cube containing 510/25 nm excitation filter, 545/40 emission filter, 525 nm dichroic (Chroma), with a measured power of 14.7 mW/mm^2^ in the imaging plane. To image Ace2N-mNeon, the filter cube contained a 470/24 nm excitation filter, 525/40 nm emission filter, 506 nm dichroic with a measured power of 18.1 mW/mm^2^ in the imaging plane. To image VARNAM, the filter cube contained 575/25 nm excitation filter, 610LP emission filter, 594 nm dichroic, with a measured power of 32.8 mW/mm^2^.

The uM Workstation was controlled by the electrophysiology software ACQ4 (RRID:SCR_016444)^62^, modified to perform fully automated experiments. To generate fluorescence/voltage curves, the membrane potential was stepped from +50 to −110 mV in 20 mV increments from a resting potential of −70 mV (0.5 s baseline, 1 s step). For current clamp recordings, a short current pulse was injected (2 nA, 2 ms) to evoke APs.

Stimulus timing, baseline fluorescence calculation, background subtraction, and photobleaching correction was performed the same way as for the field stimulation assay. To identify responsive pixels, a Mann-Whitney U test was performed between the baseline and voltage step segments of the recording. The P value criterion to identify responsive pixels was empirically set to 1e-10.

To calculate onset and decay kinetics, neurons were imaged at 3.2 kHz. Both onset and decay kinetics were fit with a double exponential equation to extract $\tau_{fast}$ and $\tau_{slow}$:

$$y_{fit}(t)=ae^{-\frac{t}{\tau_{fast}}}+be^{-\frac{t}{\tau_{slow}}}+c$$

#### Imaging and whole-cell recording in brain slices

Stereotaxic injections were made into right visual cortex (3.8 mm posterior and 3.0 mm lateral from bregma) of ~4-week-old Sst-IRES-Cre driver mice under isoflurane anesthesia. Two injections of 200 nL each of AAV2/1-syn-Flex-Voltron_585_-ST and AAV2/1-syn-Flex-Voltron2_585_-ST and were targeted to 300 and 600 μm below the cortical surface.

Four weeks later, isoflurane-anesthetized mice were transcardially perfused with ice-cold NMDG slicing solution containing (in mM): 98 HCl, 96 N-methyl-d-glucamine (NMDG), 2.5 KCl, 25 D-Glucose, 25 NaHCO_3_, 17.5 HEPES, 12 N-acetylcysteine, 10 MgSO_4_, 5 Na-L-Ascorbate, 3 Myo-inositol, 3 Na Pyruvate, 2 Thiourea, 1.25 NaH_2_PO4·H_2_O, 0.5 CaCl_2_, and 0.01 taurine. Acute 350 μm parasagittal slices containing primary visual cortex from the right hemisphere were prepared with a Compresstome (Precisionary Instruments) in ice-cold NMDG slicing solution at a slice angle of 17° relative to the sagittal plane. Slices were incubated for 10min in NMDG slicing solution at 34°C and then transferred to artificial cerebrospinal fluid (aCSF) containing in mM: 94 NaCl, 25 D-Glucose, 25 NaHCO_3_, 14 HEPES, 12.3 N-acetylcysteine, 5 Na-L-Ascorbate, 3 Myo-inositol, 3 Na Pyruvate, 2.5 KCl, 2 CaCl_2_, 2 MgSO_4_, 2 Thiourea, 1.25 NaH_2_PO_4_·H_2_0, 0.01 Taurine. All solutions were maintained under constant carbogen (95% O_2_; 5% CO_2_).

To complete fluorescent labeling of Voltron-expressing cells, 1 nM of JF_585_ was dissolved in 20 μL dimethyl sulfoxide (DMSO) and 20 μL of 20% Pluronic F-127 (w/w in DMSO). The solubilized dye was then added to 20 mL of oxygenated aCSF and incubated with the acute brain slices for one hour at room temperature, after which the slices were removed to a holding chamber (BSK 12, Scientific Systems Design) containing 500 mL oxygenated aCSF without dye. Slices were kept in this latter solution for at least one hour at room temperature prior to any experiment.

Slices were visualized using oblique (Olympus; WI-OBCD) infrared illumination using 20x or 4x objectives (Olympus). Recording pipettes were pulled from filamented borosilicate glass (Sutter Instruments) to a tip resistance of 3–8 MΩ using a DMZ Zeitz-Puller (Zeitz). Electrophysiology, image collection and subsequent analysis were performed using ACQ4. Signals were amplified using Multiclamp 700B amplifiers (Molecular Devices) and digitized at 50–200 kHz using ITC-1600 digitizers (Heka). Neurons were held in whole-cell patch clamp with an internal solution containing (in mM): 130 K-gluconate, 10 HEPES, 0.3 ethylene glycol-bis(β-aminoethyl ether)-N,N,N’,N’-tetraacetic acid (EGTA), 3 KCl, 0.23 Na_2_GTP, 6.35 Na_2_Phosphocreatine, 3.4 Mg-ATP, 13.4 biocytin, and 50 μM Cascade Blue dye.

Voltron_585_-associated fluorescence was examined using a 595 nm LED (Thorlabs) at 6.9 μW/mm^2^ power and 598/25 nm excitation and 650/54 nm emission filters (Semrock). Images were collected by sampling a 675μm x 137μm region of the slice with a digital sCMOS camera (Hamamatsu; Flash 4.0 V2) at 500 Hz and 4×4 pixel binning. Image analysis was performed by custom routines written in Python (RRID:SCR_008394). For each camera frame, average fluorescence intensity over an elliptical region of interest (ROI) over neuropil adjacent to a cell was subtracted from an identically shaped region containing the cell itself. Synthetic post-synaptic potentials (synPSPs) of −15mV to +15mV in 5mV increments were created through current injection and repeated for a total of 10 trials per cell. Raw fluorescence signals were converted to percentage change in fluorescence ($\text{ΔF}/{\text{F}}_{0}$) relative to a baseline trace generated by a least-squares regression line fit to a 40ms period prior to the synPSP. Sensitivity index (d’)^63^ was calculated for each synPSP for each cell by determining the average $\text{ΔF}/{\text{F}}_{0}$ of a 10ms “noise” window prior to the onset of the synPSP and a “signal” window centered over the fluorescence peak (from 4 ms to 14 ms after the onset of the change in membrane potential) as follows:

$$d^{\prime }=\left(\mu_{S}-\mu_{N}\right)/\sqrt{\frac{1}{2}\left({\sigma_{S}}^{2}+{\sigma_{N}}^{2}\right)}$$

where ${\text{μ}}_{\text{s}}$ and ${\text{σ}}_{\text{s}}$ are the mean and standard deviation, respectively, of the signal window across the 10 individual trials, and ${\text{μ}}_{\text{N}}$ and ${\text{σ}}_{\text{N}}$ are the mean and standard deviation, respectively, of the noise window across the 10 trials. This is also expressed as a single value independent of the experimental condition by reporting the slope of a linear regression for each cell across all the voltage steps as d’/mV.

#### Simultaneous voltage imaging and optogenetic stimulation in brain slices

Voltron2 and Channelrhodopsin2 (ChR2) were expressed throughout the motor cortex using injections of a mixture of (1) rAAVretro-hSyn-Cre-WPRE (2×10^9^ GC.; Addgene #105553-AAVrg), (2) AAV1-Syn-FLEX-Voltron2_585_-WPRE (1×10^9^ GC), (3) AAV8-Syn-ChR2(H134R)-GFP (3×10^8^ GC; Addgene #58880-AAV8), and (4) 0.05% Trypan Blue in 1 μL of sterile PBS into the lateral ventricle of C57Bl/6N mice (Charles River) at postnatal day 1^64^. At least 14 days following virus injection, mice were transcardially perfused with 15 mL of chilled and carbogen-bubbled (95% O_2_/5% CO_2_) NMDG aCSF solution (in mM: 92 NMDG, 2.5 KCl, 1.25 NaH_2_PO_4_, 30 NaHCO_3_, 20 HEPES, 25 glucose, 2 thiourea, 5 Na-ascorbate, 3 Na-pyruvate, 0.5 CaCl_2_·4H_2_O and 10 MgSO_4_·7H_2_O, pH 7.3–7.4, 300–310 mOsm). Acute slices through motor cortex were made in chilled NMDG aCSF with constant bubbling^65^. Following re-introduction of sodium in 37°C NMDG aCSF, slices were transferred to a holding chamber containing 25 nM JF_585_ dye in 5 mL bubbled, room temperature HEPES aCSF buffer (in mM: 92 NaCl, 2.5 KCl, 1.25 NaH_2_PO_4_, 30 NaHCO_3_, 20 HEPES, 25 glucose, 2 thiourea, 5 Na-ascorbate, 3 Na-pyruvate, 2 CaCl_2_·4H_2_O and 2 MgSO_4_·7H_2_O, pH 7.3–7.4, 300–310 mOsm). Slices were incubated in dye solution for one hour, and moved to fresh HEPES aCSF for one hour to wash out excess dye. Experiments were performed at room temperature in HEPES aCSF solution. Whole-cell recordings were made using filamented glass pipettes (Sutter #BF150–86-10) pulled to 3–8 MΩ resistance (Sutter P-1000 Micropipette Puller), and intracellular recording buffer containing (in mM) 145 K-Gluconate, 10 HEPES, 1 EGTA, 2 Mg-ATP, 0.3 Na_2_-GTP, and 2 MgCl_2_ (pH 7.3, 290–300 mOsm). A patch-clamp headstage (Molecular Devices #1-CV-7B) mounted on a motorized 4-axis Siskiyou MX7600 manipulator, and Axon Instruments MultiClamp 700b amplifier were used for all recordings.

Imaging was performed using a custom-built confocal microscope at a frame rate of 458 Hz using a 16X/0.8 NA water-immersion objective lens (Nikon CFI75 LWD 16X W). High frame rates were achieved using a system similar to that described previously^66^ but with a 128-facet polygonal scanner (Cambridge Technology SA34) substituted for the x-axis scanner. Voltron2_585_ was excited with a 561 nm laser diode (Vortran Stradus). The time-averaged irradiance at the sample was 33 mW/mm^2^ and fluorescence was collected with a dichroic mirror and emission filter (Chroma T570lpxr and ET570lp), detected with a silicon photomultiplier (Hamamatsu S14420–1550MG, V_BIAS_ = 50 V) and amplified on-board with a custom circuit (https://github.com/tweber225/simple-sipm). A blue LED (Thorlabs M470L4) was used to provide full-field ChR2 stimulation. The LED was filtered and coupled into the confocal beam path with an excitation filter and dichroic mirror (Thorlabs MF475–35 and DMLP505R). Additionally, the LED was attenuated such that the desired irradiance levels (10–50 μW/mm^2^) were within the analog control range of the LED driver (Thorlabs LEDD1B). Image acquisition and stimulus timing were managed with ScanImage^67^ and WaveSurfer (https://wavesurfer.janelia.org/).

#### Lattice lightsheet imaging in zebrafish

*In vivo* light sheet microscopy of zebrafish was performed as previously described^68^. Briefly, zebrafish transgenic lines expressing soma-tagged Voltron (Tg[vglut2a:Gal4; UAS:Voltron-ST]) and Voltron2 (Tg[vglut2a:Gal4;UAS:Voltron2-ST]) were generated. At three dpf, fish were incubated in a water solution containing 3 μM JF_552_ for 2 h. The fish at 4 to 6 dpf were then paralyzed by a-bungarotoxin (1 mg/mL) and mounted in low melting point agarose for imaging. The custom microscope used for imaging was described previously^68^. Here it was used without the adaptive optics (AO) subsystem since optical aberration was negligible in the structure we imaged. A 740 nm thick light sheet was created from a 560 nm laser source using a multi-Bessel lattice with an outer and inner NA of 0.38 and 0.36, respectively, for a measured power of 100 μW at the back pupil of the excitation objective. Single-plane imaging was performed at an effective 108 × 108 nm XY resolution, with an FOV of 256×512 pixels, at a framerate of 400 Hz. Approximately 4–10 Voltron-expressing neurons were present in each field of view. Fluorescent signal was recorded for 5 minutes. For analysis, the automated voltage imaging analysis package Volpy was used^69^. To perform an unbiased comparison of Voltron_552_-ST and Voltron2_552_-ST populations, every spiking cell detected by Volpy was included in the analyzed dataset, irrespective of AP amplitude. $\text{ΔF}/{\text{F}}_{0}$ and SNR for each cell were calculated by Volpy. For SNR calculations, the noise was defined as the standard deviation of the residual after subtracting spike and subthreshold components, as detected by Volpy.

#### Voltron imaging in adult flies

Experiments were performed as described previously^19^. Briefly, crosses of Voltron (*UAS-IVS-syn21-Ace2NHalo-p10* Su(Hw)attP8) or Voltron2 (*UAS-IVS-syn21-Ace2N(A122D)Halo-p10* Su(Hw)attP8) reporters with split Gal4 drivers were raised on standard cornmeal food supplemented with all-trans-retinal (0.2 mM before eclosion and then 0.4 mM). 2- to 10-day old female progeny were collected for experiments. To prepare the fly for imaging, a small hole was dissected in the head capsule, and air sacs and fat tissue were removed but we did not intentionally remove the perineural sheath. The exposed brain was then bathed in a drop (~200 μL) of dye-containing saline (1 μM for JF_552_-Halotag ligand) for 1 hr. Saline contained (in mM): NaCl, 103; KCl, 3; CaCl_2_, 1.5; MgCl_2_, 4; NaHCO_3_, 26; N-tris(hydroxymethyl)methyl-2-aminoethanesulfonic acid, 5; NaH_2_PO_4_, 1; trehalose, 10; glucose, 10 (pH 7.3 when bubbled with 95% O_2_ and 5% CO_2_, 275 mOsm). The dye was then washed-out by rinsing three times with ~10 mL of fresh saline each time over a 1-hr period. Imaging was performed on a widefield fluorescence microscope (SOM, Sutter Instruments) equipped with a 60x, 1.0 NA water-immersion objective (LUMPlanFl/IR; Olympus) and an sCMOS camera (Orca Flash 4.0 V3, Hamamatsu). Images were acquired at 800 Hz with 4×4 binning through the Hamamatsu imaging software (HCImage Live; RRID:SCR_015041). For JF_552_, illumination was provided by a 561-nm LED (SA-561–1PLUS, Sutter) with an excitation filter (FF01–549/12–25, Semrock); intensity at the sample plane was 2–11 mW/mm^2^ for typical recordings. Emission was separated from excitation light using a dichroic mirror (FF562-Di03–25×36, Semrock) and an emission filter (FF01–590/36–25, Semrock). We found that JF_552_ allows for longer-duration imaging compared with JF_549_ and JF_525_, which we used previously^19^. At the aforementioned illumination levels, spiking activity was detectable for over 20 minutes in PPL1-γ1pedc and over 10 minutes in MBON-γ1pedc>α/β.

For MBON-γ1pedc>α/β imaging experiments, both left and right hemispheres were sampled, while for PPL1-γ1pedc, whose axons project bilaterally, only one hemisphere was imaged. Each experiment at one illumination level consists of a recording of 15 s. Data were analyzed with custom-written scripts in MATLAB (RRID:SCR_001622). Regions of interest (ROIs) corresponding to the γ1 region were manually selected, and the mean pixel intensity within the ROI was calculated. The raw fluorescence trace was de-trended by median filtering with a 50 ms time window. ${\text{F}}_{0}$ was calculated from the filtered trace as the mean over the first 1 s of imaging session. Spike sorting and SNR quantification were performed on the de-trended trace. Spikes were automatically detected by finding local minima and verified by visual inspection. SNR was quantified as peak amplitude over the standard deviation of the trace excluding a 50 ms time window around any spikes.

#### Imaging Parvalbumin (PV) interneurons in mouse hippocampus

Hippocampal PV neuron imaging was performed using adult PV-Cre mice (RRID:IMSR_JAX:008069). The imaging window was implanted using procedures similar to those previously described^70^. In short, a circular craniotomy (3 mm diameter) was made centered at 2.0 mm caudal and 2.0 mm lateral to bregma. The surface of the CA1 region was exposed by gently removing the overlying cortex with aspiration. AAV2/1-syn-FLEX-Voltron_552_-ST (Voltron_552_; 4 mice) and either AAV2/1-syn-FLEX-Voltron2_552_-ST (2 mice) or AAV2/1-CAG-FLEX-Voltron2_552_-ST (Voltron2_552_, 3 mice) virus was diluted to 1.4×10^13^, 4.1×10^13^ and 8.26×10^11^ GC/mL, respectively. Diluted viruses were injected at four locations (separated by 700 μm, 50 nL per location) at a depth of 200 μm from CA1 surface (injection rate, 1 nL/s). The imaging window (constructed by gluing a 3 mm diameter cover glass to a stainless steel cannula of 3 mm diameter and 1.5 mm height) was placed onto the hippocampus and glued to the skull using super-bond C&B (Sun Medical). A titanium head bar was glued to the skull for head fixation during imaging.

Imaging experiments started 3 weeks after surgery. 100 nM of JF_552_ were dissolved in 20μL of DMSO (Sigma) and diluted in 20 μL Pluronic^™^ F-127 (20% w/v in DMSO, P3000MP, Invitrogen) and in 80 μL PBS. The dye solution was delivered using retro-orbital injection^71^ with a 30 gage needle. Three hours after dye injection, animals were placed under the microscope and labeled PV+ cells (47 – 137 μm deep) were illuminated using a 532 nm laser (Opus 532, Laser Quantum) through an excitation filter (FF02–520-28, Semrock). Fluorescence was collected using a 16X/0.8 NA objective (Nikon), separated from excitation light using a dichroic mirror (540lpxr, Chroma) and an emission filter (FF01–596/83, Semrock), and imaged onto a CMOS camera (DaVinci-1K, RedShirt) using a 50 mm camera lens (Nikkor 50 mm f1.2, Nikon) as the tube lens. For patterned illumination, the laser beam was expanded using a pair of lenses (C280TMD-A and AC254–150-A, Thorlabs) and directed to a digital micromirror device (DMD; V7000, ViALUX). The DMD was imaged to the sample using an 80 mm lens (AC254–080-A, Thorlabs) and the microscope objective. A reference image of labeled cells was first acquired using widefield illumination. Bright and in-focus neurons were selected manually and their coordinates were used to generate an illumination mask consisting of 64 μm diameter discs centered on each selected cell. The illumination intensity was ~70–140 mW/mm^2^ (i.e. ~0.22 – 0.45 mW per cell) at the sample plane. Images (190 × 160 pixels, corresponding to an area of 1.4 × 1.2 mm) were collected at 2 kHz using Turbo-SM64 software (Sci-Measure) for three minutes (360,000 images).

Brain motion was corrected using rigid registration. The fluorescence F(t) of each cell was measured by averaging pixel values within a 10-pixel region covering the cell body. To correct for bleaching and other slow fluctuations, a baseline fluorescence trace ${\text{F}}_{0}(\text{t})$ was computed from F(t) by a moving average with 1 s windows. Since Voltron fluorescence decreases with membrane depolarization, we define $\text{ΔF}/{\text{F}}_{0}(\text{t})=(-(\text{F}(\text{t})-{\text{F}}_{0}(\text{t})))/\left({\text{F}}_{0}(\text{t})\right)$ as an estimate of membrane potential. To detect APs, a high pass filtered version of $\text{ΔF}/{\text{F}}_{0}$, ${\left(\text{ΔF}/{\text{F}}_{0}\right)}_{\text{hp}}$, was computed by subtracting a median-filtered (5 ms window) $\text{ΔF}/{\text{F}}_{0}$. Positive peaks of the ${\left(\text{ΔF}/{\text{F}}_{0}\right)}_{\text{hp}}$ trace were detected and considered as candidate spike locations (with ${\text{t}}_{\text{k}}$ and ${\text{p}}_{\text{k}}$ being the locations and the amplitudes, respectively, of the k^th^ candidate peak). To choose a threshold, the distribution of ${\text{p}}_{\text{k}}$, P(x), was estimated by kernel density method (‘ksdensity’ function in MATLAB). The same procedure was applied to the inverted ${\left(\text{ΔF}/{\text{F}}_{0}\right)}_{\text{hp}}$ trace to detect ‘noise’ peaks, and the amplitudes of those peaks were used to construct a noise distribution, ${\text{P}}_{\text{noise}}(\text{x})$. The distribution of spike amplitudes was estimated as $\text{S}(\text{x})=\text{P}(\text{x})-{\text{P}}_{\text{noise}}(\text{x})$, and a threshold value *th1* was chosen at the location where $\text{S}(th1)={\text{P}}_{\text{noise}}(th1\text{)}$ in order to minimize the sum of type I and type II error. This approach works well in cells with good signal to noise ratio (SNR), but in low SNR cells it often leads to substantial false positive detections. We estimated the number of false positive detections (*nFP*), at any given threshold value, by counting the number supra-threshold ‘noise’ peaks in the inverted ${\left(\text{ΔF}/{\text{F}}_{0}\right)}_{\text{hp}}$ trace. If *nFP* at *th1* exceeds 18 over the 180 s recording period (i.e. false positive rate > 0.1 Hz), the threshold was replaced by a higher value, *th2*, that allowed a maximum of 18 false positive detections. Candidate peaks larger than the threshold were used for an initial estimate of spike times, and segments of the ${\left(\text{ΔF}/{\text{F}}_{0}\right)}_{\text{hp}}$ trace around these peaks were averaged to generate an initial estimate of the AP waveform, AP(t). Since AP waveforms exhibit finite rise and decay times, the occurrence of a spike interferes with the detection of spikes within its immediate neighborhood. To correct for this effect, if a candidate peak ${\text{p}}_{\text{i}}$ was surrounded by a larger peak ${\text{p}}_{\text{j}}$ within ± 2 ms, its amplitude was corrected by assuming that a spike occurred at ${\text{t}}_{\text{j}}$ and by subtracting the contribution of that spike, i.e. ${\text{p}}_{\text{i},\text{corrected}}={\text{p}}_{\text{i}}-\text{AP}\left({\text{t}}_{\text{i}}-{\text{t}}_{\text{j}}\right)$. This procedure was used to correct the amplitudes of all candidate peaks. Finally, a candidate peak was detected as an AP if its corrected amplitude exceeded the above mentioned threshold.

To quantify the recording quality and the fidelity of spike detection, we first estimated the spike amplitude A by averaging the amplitudes of all detected spikes. The noise of the recording σ was estimated as the standard deviation of the ${\left(\text{ΔF}/{\text{F}}_{0}\right)}_{\text{hp}}$ trace excluding regions 2 ms before and 4 ms after each detected spike. The signal to noise ratio was measured for each cell as SNR=A/σ. A cell was included into our analysis if (1) its SNR exceeded 5, (2) the number of detected spikes in the cell exceeded 90 (i.e. spike rate > 0.5 Hz), (3) less than 1% of detected spikes had an inter-spike-interval less than 2.0 ms, and (4) the half-width of the spike waveform was shorter than 0.85ms. To compare the density of labeled neurons, a z-stack of images was acquired at the end of the recording session and cell bodies in a 1280 ×1280×200 μm^3^ volume were counted manually.

#### Imaging in mouse visual cortex

Layer 2/3 pyramidal neurons in the visual cortex of mice (C57BI/6NCrl; Charles River Laboratories) were sparsely labeled with the indicators – either Voltron_525_-ST or Voltron2_525_-ST. We prepared four mice per group. In an anesthetized mouse (isoflurane in pure oxygen; 4% for induction, 1–2% for maintenance), we first glued a ring-shaped titanium headbar to the skull of the animal using a gel-form cyanoacrylate and then fully closed the skin around the headbar. A craniotomy (4.5 mm in diameter) was drilled over the left parietal cortex, centered on −2.5 mm lateral, +0.5 mm anterior from lambda (visual cortex). Using beveled, pulled-glass capillaries (tip size <12 μm), we injected a mixture of two viruses: high-titer AAV carrying the cassette for conditional expression of the voltage indicator (AAV2/1-syn-FLEX-Voltron-ST or AAV2/1-syn-FLEX-Voltron2-ST; titer 10^12^ GC/mL) and low-titer AAV carrying the transcription permissive signal (AAV9-CamKIIa-Cre; titer 10^8^ GC/mL). Six to eight 40 nL injections at a depth of 150 μm were performed in each mouse. The craniotomy was coversliped and the cranial window was secured using cyanoacrylate. A standard analgesia protocol (ketoprofen) followed. Approximately seven weeks after surgery, the animal was prepared for imaging. One day prior to imaging, JF_525_ dye was administered intravenously. To prepare the JF dye for injection, 100 nM of lyophilized JF_525_ was dissolved in 20 μL of DMSO, 20 μL Pluronic F-127 (20% w/v in DMSO), and 60–80 μL of PBS. Mice were briefly anesthetized and 100 μL of the dye solution was injected into the retro-orbital sinus of the right eye using a 30-gauge needle. We used the same design of wide-field fluorescence microscopy with structured illumination as previously described ^19^. Illumination was delivered using a 525 nm LED (Mightex, LCS-0525–60-22) and shaped using a digital mirror device (Texas Instruments, LightCrafter). The microscope was equipped with a water immersion objective (20X, NA 1.0, Olympus XLUMPLFLN) and a CMOS camera (Hamamatsu Orca Flash v3). Excitation and emission were separated using a standard filter cube (Chroma 49014; excitation 530/30, dichroic 550, emission 575/40). The illumination was restricted to single neurons using a DMD. The illuminated spot was 80 μm in diameter and the intensity was kept at 18.5 mW/mm^2^ in the sample plane. Small fields of view (40 μm X 40 μm) containing single neurons were typically captured. The native 2048×2048 resolution of the camera was binned by a factor of 4. During imaging, we recorded only from neurons that produced at least ~120 photons per frame and per pixel as this was expected to lead to approximately 1% standard deviation of the raw signal (quantum efficiency of the camera 82%, neuron covered with ~100 pixels, noise dominated by shot noise). Three-minute time series at 500 Hz were captured for most of the recordings; one minute at 1 kHz was used only for comparison of AP-related fluorescence changes. Mice were imaged fully awake without any visual stimulation.

To process the recordings, we first removed the in-plane motion artifacts using the fast rigid registration algorithm NoRMCorre ^72^. Neurons were then segmented manually. The signal was taken as the mean intensity over the region of the interest. The *in vitro* data showed a substantial difference in brightness of the two indicators. Since voltage-independent background autofluorescence (presumed to also be independent of the chosen indicator) would comprise different fractions of the signal and decrease the observed relative fluorescence changes, we subtracted the mean intensity of the neuropil surrounding each particular neuron from its signal (${\text{I}}_{\text{n}}$). To detrend the signal and extract the fluorescence changes related to both APs and slower membrane voltage changes (EPSPs, oscillations), we calculated a baseline (${\text{B}}_{5\text{s}}$) using a 5s median filter. The $\text{ΔF}/{\text{F}}_{0}$ trace was then defined as $\text{ΔF}/{\text{F}}_{0}=100*\left(\ln-{\text{B}}_{5\text{s}}\right)/{\text{B}}_{5\text{s}}$. To extract only the AP-related fluorescence spikes, we calculated another baseline (${\text{B}}_{20}$) using a 20 ms median filter; $\text{ΔF}/{\text{F}}_{\text{APs}}=100*\left({\text{I}}_{\text{n}}-{\text{B}}_{20\text{ms}}\right)/{\text{B}}_{20\text{ms}}$. We estimated the noise directly from the $\text{ΔF}/{\text{F}}_{\text{APs}}$ trace. Based on the fact that the AP-related spikes are all negative-going and APs are generally sparse, positive values of the trace $\text{ΔF}/{\text{F}}_{\text{APs}}$ can be considered as noise. We removed all negative data points and then randomly assigned positive/negative signs to the rest of the points. We calculated the standard deviation of these values (${\text{SD}}_{\text{noise}.}$) for each neuron and set it as a threshold to detect spikes; $\text{THR}=-4^{*}{\text{SD}}_{\text{noise}.}$. If the threshold was crossed at two neighboring time points, such doublet was considered as a single AP, the time point with higher amplitude was chosen by the algorithm and the spike was ascribed to this time point. Using four standard deviations leads to false positivity rate of 1–2 false spikes per minute in our recordings.

To detect the periods of 3–5 Hz oscillations, we applied a bandpass filter (3–5Hz, MATLAB [bandpass]) to the $\text{ΔF}/{\text{F}}_{0}$ trace and then detected the pronounced oscillations as outliers from the values’ variance. The bandpassed trace was thresholded by 3*MAD (median absolute deviations) using the MATLAB function [isoutlier]. Absolute values of these outliers were averaged for each neuron and later averaged over all neurons in both groups. Two-tailed statistical tests were used unless indicated otherwise.

#### Imaging in mouse anterior lateral motor cortex

NDNF-Cre mice (RRID:IMSR_JAX:028536) were used for imaging layer 1 neurons (3 females, 4 males; 10–14 weeks old at the time of the window surgery). NDNF-Cre mice were injected with 30 nL of AAV2/1-syn-FLEX-Voltron-ST (titer, 2×10^12^ GC/mL) or AAV2/1-syn-FLEX-Voltron2-ST (titer, 10^11^ GC/mL) at 8–12 injection sites 200 μm deep (injection rate, 1 nL/s). Cranial windows (2.5 mm diameter) were implanted over the injection sites in the anterior lateral motor cortex (ALM), centered on −1.5 mm lateral, +2.5 mm anterior from bregma. Four to nine weeks later JF_552_ dye was injected into the retro-orbital sinus. Imaging was done 1 to 2 days after dye injection, with subsequent dye injections and imaging 1 to 6 weeks after the first imaging session. To prepare the JF dye for injection, 100 nM of lyophilized JF_552_ were dissolved in 20 μL of DMSO, 20 μL Pluronic F-127 (20% w/v in DMSO), and 60 μL of PBS (final dye concentration 1 μM). Mice were anesthetized with 2–3% isoflurane and 100 μl of the dye solution was injected into the retro-orbital sinus of the right eye using a 29 gauge needle^71^. A widefield fluorescence microscope equipped with a water immersion objective (20X, NA 1.0, Olympus XLUMPLFLN) was used for imaging. Illumination was delivered using a 525 nm LED (Mightex, LCS-0552– 60-22); intensity at the sample, <20 mW/mm^2^. A filter set (530/55 nm excitation, 625/90 nm emission, 561nm-LP dichroic mirror) was used for fluorescence imaging of Voltron and Voltron2. Images were collected using a sCMOS camera (Hamamatsu Orca Flash 4.0 v3) at a frame rate of 400 Hz. A 0.55X magnification camera tube was placed between the objective and the camera. The size of the field of view was 1060 μm X 265 μm. The pixel resolution was 2.08 μm/pixel. Mice were water restricted and imaged awake while performing a delayed response licking task.

To identify neuronal activity and spatial structure from Voltron recordings, we used an iterative spatial and temporal filtering approach (Volpy). Initial ROIs (Fig. 7p) were manually drawn and used as input to Volpy. To determine whether a neuron should be used for further analysis we employed two QC metrics. The first metric is SNR, defined as the spike amplitude divided by the standard deviation of the high-frequency components of the trace:

$$SNR=\frac{\frac{1}{n}\sum_{t\epsilon S_{k}}X_{k}(t)}{\left\|X_{K}-median\left(X_{k}\right)\right\|}$$

where $S_{k}$ is the set of spike times and n is the number of detected spikes for the neuron. Neurons with SNR<3 were excluded. The second metric is the overlap of the spatial filter generated by Volpy with the initial manually-drawn ROI. For each neuron, if the center of the computed spatial filter was not within the initial ROI (suggesting that the signals in the initial ROI are mostly background noise), the neuron was excluded. For the neurons that passed QC, spike $\text{ΔF}/{\text{F}}_{0}$ (Fig. 7s) was calculated using the spike times and $\text{ΔF}/{\text{F}}_{0}$ trace estimated by Volpy. For each cell, we selected spikes that occurred <20 ms after the previous spike. We calculated the amplitude of each such spike as the difference between the $\text{ΔF}/{\text{F}}_{0}$ at the peak of the spike and the trough between the spike and the previous spike. The spike $\text{ΔF}/{\text{F}}_{0}$ for a cell was the average amplitude of all spikes detected for that cell.

### Quantification and Statistical Analysis

All statistical analysis were performed using MATLAB, Python, or Graphpad Prism.

## Supplementary Material

Supplemental information

Table S1 Table S1: Screening results of field stimulation assay on Voltron point mutants. Related to Fig. 1. Normalization performed to in-plate Voltron controls.

Table S2 Table S2: Screening results of field stimulation assay on Voltron combo mutants. Related to Fig. 1. Normalization performed to in-plate Voltron controls.

Table S3 Table S3: Combo variants containing the A122D mutation, arranged by the number of mutations. Related to Fig. 1. Data aggregated from Supplementary Table 2.

## Figures and Tables

**Table:** Key resources table

REAGENT or RESOURCE	SOURCE	IDENTIFIER
**Bacterial and virus strains**		
rAAVretro-hSyn-Cre-WPRE	Addgene	Addgene: 105553-AAVrg
AAV1-Syn-FLEX-Voltron2-WPRE	This paper	N/A
AAV8-Syn-ChR2(H134R)-GFP	(Boyden et al. 2005)	Addgene: 58880-AAV8
AAV2/1-syn-FLEX-Voltron-ST	(Abdelfattah et al. 2019)	Addgene: 119036-AAV1
AAV2/1-syn-FLEX-Voltron2-ST	This paper	N/A
**Experimental models: Organisms/strains**		
Mouse: B6;129P2-*Pvalb*^*tm1(cre)Arbr*^/J	The Jackson Laboratory	RRID:IMSR_JAX:008069
Mouse: B6.Cg-*Ndnf*^*tm1.1(folA/cre)Hze*^/J	The Jackson Laboratory	RRID:IMSR_JAX:028536
Mouse: C57BL/6	Charles River	N/A
Rat: Time pregnant rats	Charles River	N/A
Fly: UAS-IVS-syn21-Ace2NHalo-p10 Su(Hw)attP8	This paper	N/A
Fly: UAS-IVS-syn21-Ace2N(A122D)Halo-p10 Su(Hw)attP8	This paper	N/A
Zebrafish: Tg[vglut2a:Gal4; UAS:Voltron-ST]	This paper	N/A
Zebrafish: Tg[vglut2a:Gal4; UAS:Voltron2-ST]	This paper	N/A
**Recombinant DNA**		
pAAV-syn-FLEX-Ace2N-4AA-mNeon-ST A122D WPRE	Addgene	Addgene: 172908
pGP-pcDNA3.1 Puro-CAG-Voltron2	Addgene	Addgene: 172909
pGP-pcDNA3.1 Puro-CAG-Voltron2-ST	Addgene	Addgene: 172910
pGP-CAG-Ace2N-4AA-mNeon A122D-WPRE-bGH-polyA	Addgene	Addgene: 172911
pGP-CAG-Ace2N-4AA-mNeon-ST A122D-WPRE-bGH-polyA	Addgene	Addgene: 172912
pGP-CAG-VARNAM A122D WPRE-bGH-polyA	Addgene	Addgene: 180486
**Software and algorithms**		
ilastik	(Berg et al. 2019)	RRID:SCR_015246
Acq4	(Campagnola et al. 2014)	RRID:SCR_016444
Python	Python Software Foundation	RRID:SCR_008394
Volpy	(Cai et al. 2021)	N/A
HCImage Live	Hamamatsu Photonics	RRID:SCR_015041
MATLAB	Mathworks	RRID:SCR_001622
NoRMCorre	(Pnevmatikakis and Giovannucci 2017)	N/A
**Other**		
Inverted microscope	Olympus	IX-81
EMCCD camera	Andor	Ixon Ultra DU897
Optomask	Cairn Research	N/A
Electrical stimulator	Grass Instruments	S-48
Automated uM Workstation	Sensapex	N/A
Multiclamp 700B	Molecular Devices	N/A
A/D converter	HEKA	ITC-1600
sCMOS camera	Hamamatsu	Flash 4.0 V2/V3
CMOS camera	RedShirt	DaVinci-1K
Digital Micromirror Device	ViALUX	V-7000
Digital Micromirror Device	Texas Instruments	LightCrafter

## Highlights

- Voltron2 is an improved version of the chemigenetic voltage indicator Voltron
- A point mutation enhances the sensitivity of several rhodopsin-based sensors
- Voltron2 was tested *in vivo* in flies, fish and mice
