# Supplementary material for: Sensitivity optimization of a rhodopsin-based fluorescent voltage indicator
Source: Neuron. Author manuscript; Available in PMC 2024 May 17. (PMC10280807; doi:10.1016/j.neuron.2023.03.009)
Supplement: Supplemental information [file NIHMS1883979-supplement-Supplemental_information.pdf]

Supplemental information

Sensitivity optimization of a rhodopsin-based fluorescent voltage indicator

Ahmed S Abdelfattah, Jihong Zheng, Amrita Singh, Yi-Chieh Huang, Daniel Reep, Getahun Tsegaye, Arthur Tsang, Benjamin J Arthur, Monika Rehorova, Carl VL Olson, Yichun Shuai, Lixia Zhang, Tian-Ming Fu, Daniel E Milkie, Maria V Moya, Timothy D Weber, Andrew L Lemire, Christopher A Baker-, Natalie Falco, Qinsi Zheng, Jonathan B Grimm, Mighten C Yip, Deepika Walpita, Martin Chase, Luke Campagnola, Gabe J. Murphy, Allan M Wong, Craig R Forest, Jerome Mertz, Michael N Economo, Glenn Turner, Minoru Koyama, Bei-Jung Lin, Eric Betzig,, Ondrej Novak, Luke D Lavis, Karel Svoboda, Wyatt Korff, Tsai-Wen Chen, Eric R Schreiter, Jeremy P Hasseman, Ilya Kolb

Supplementary Figures

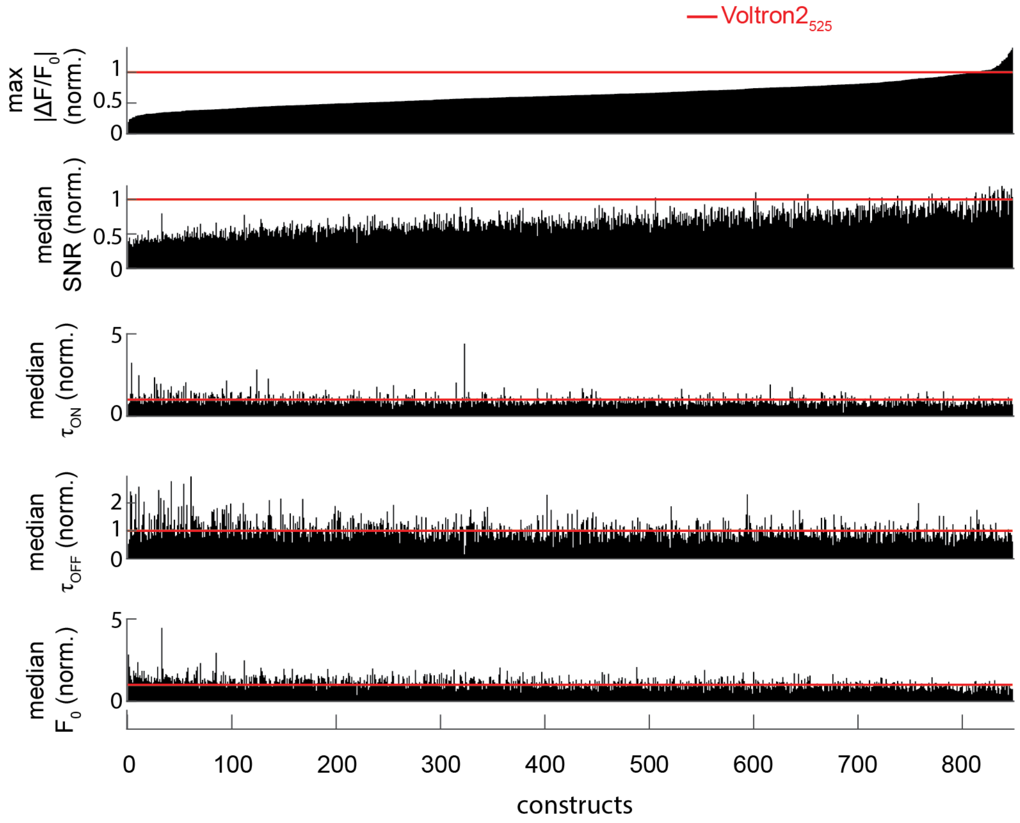

Figure S1. Field stimulation assay results of combo mutations, ranked by maximum $|\Delta F/F_0|$ for each variant, normalized to in-plate Voltron2$_{525}$ controls. Related to Fig. 1.

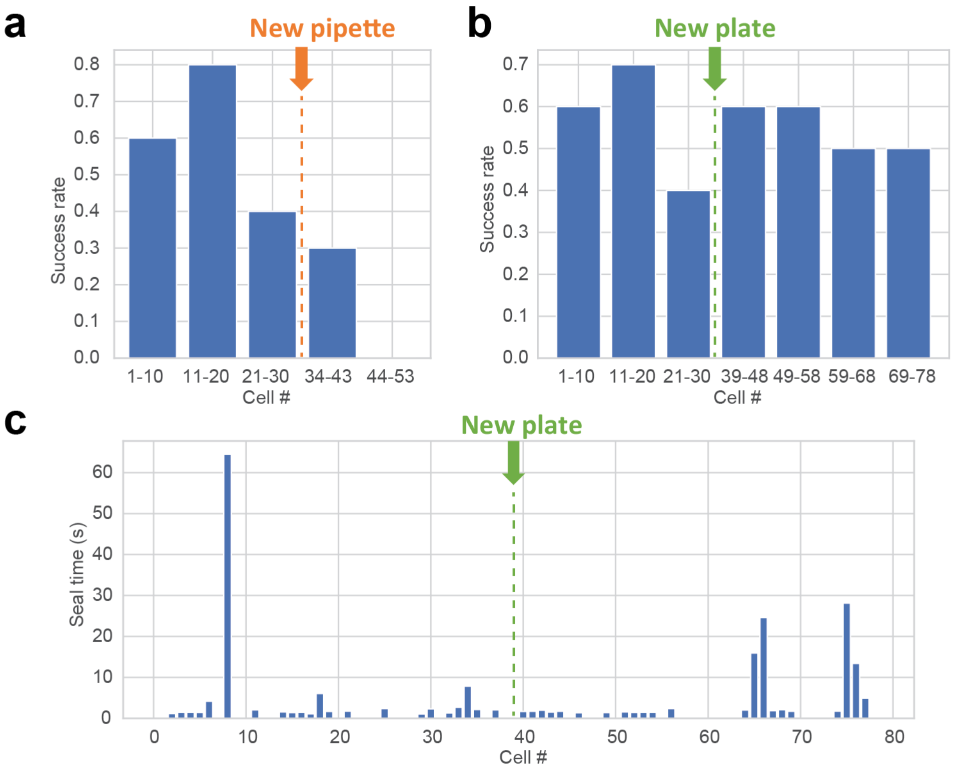

Figure S2. Pipette cleaning with the uM Workstation. Related to Fig. 2.

a. Whole-cell recording success rate with pipette cleaning after every recording. The pipette was replaced after 30 recordings, but the success rate did not improve. b. Whole-cell recording success rate with a single reused pipette. A new plate of neurons was used after the 30th cell, causing the success rate to improve. c. Time to form a Gigaohm seal over multiple cells using a single pipette. A blank entry indicates that the gigaseal was unsuccessful. A new plate of neurons was used after the 38th cell.

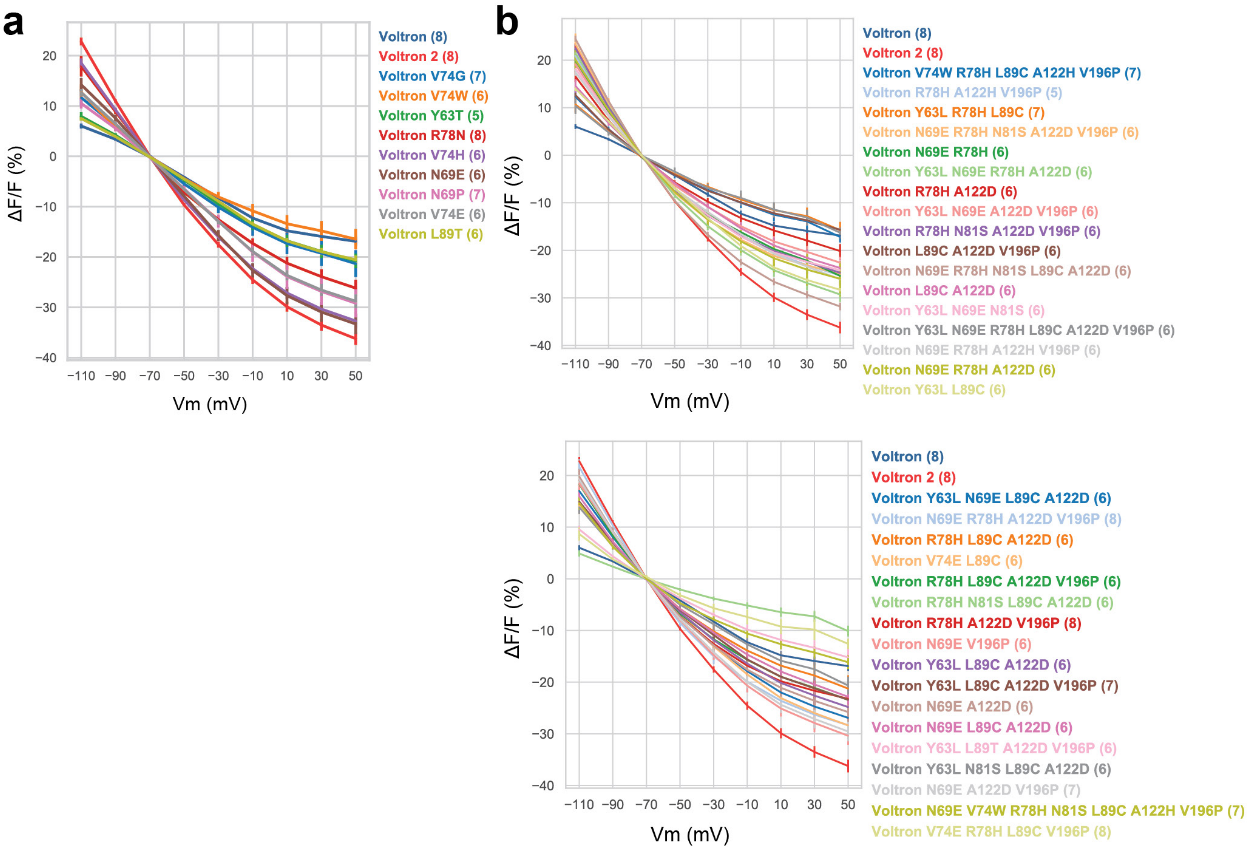

Figure S3. Patch-clamp characterization of SSM and combo Voltron mutants. Related to Fig. 2.

Peak fluorescence response to voltage steps from -70 mV of (a) the top SSM mutants and (b) the top combo mutants from the field stimulation assay, with Voltron and Voltron2 traces (reproduced from Fig. 2) superimposed for reference. Number in parentheses indicates the number of neurons assayed. All sensor mutants were conjugated to JF_{525} dyes for these experiments ($Voltron_{525}$). Values represent mean ± s.e.m.

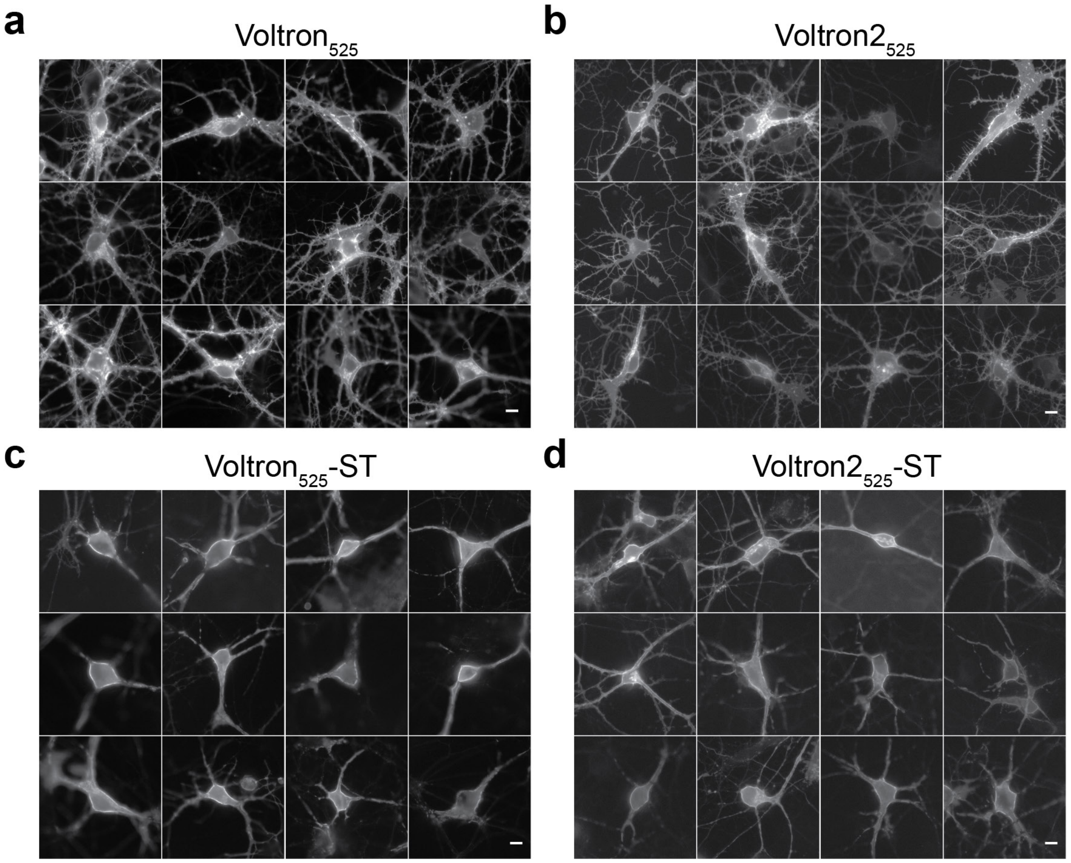

Figure S4. Representative fluorescent images of cultured hippocampal neurons expressing (a) Voltron$_{525}$, (b) Voltron2$_{525}$, (c) Voltron$_{525}$-ST, and (d) Voltron2$_{525}$-ST. Related to Fig. 2.

Images were taken with 15 mW/mm^2 light power and 30 ms exposure time. Dynamic ranges of images were rescaled for clarity. Scale bar: 10 μm

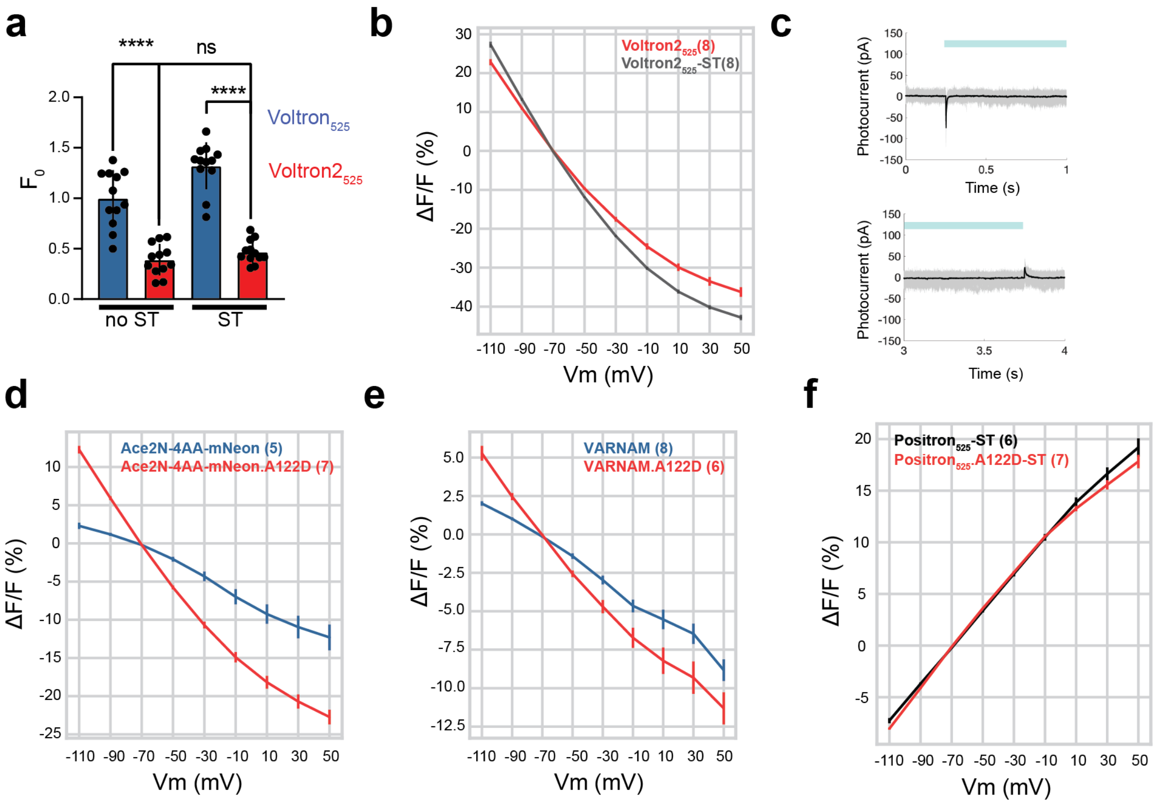

Figure S5. Brightness and patch-clamp characterization of Ace2-based GEVIs. Related to Fig. 2.

a. Baseline fluorescence of non soma-tagged (no ST) and soma-tagged (ST) Voltron$_{525}$ and Voltron2$_{525}$ (mean±s.d.; n=12 neurons for each, from single transfection; ****: p<0.0001; n.s.: p=0.80; one-way ANOVA followed by Tukey's multiple comparison test). b. Fluorescence response to voltage steps of Voltron2$_{525}$-ST, compared to Voltron2$_{525}$ (reproduced from Fig. 2). Number in parentheses indicates the number of neurons assayed. Values represent mean ± s.e.m. c. Photocurrent measurements from rat hippocampal neurons in culture expressing Voltron2, labeled with JF$_{525}$. Blue bar denotes time of light illumination (508 nm–522 nm) at an irradiance of 80 mW mm^{-2}. Experiment was 4s long and light was on between 0.25s to 3.75s. Time between 1s-3s not shown. Steady-state photocurrent for Voltron2 is negligible: −0.3 ± 2 pA, (mean ± s.d., N = 5 cells). d. Peak fluorescence response to voltage steps from -70 mV of Ace-4AA-mNeon.A122D, with Ace-4AA-mNeon as control. e. Peak fluorescence response to voltage steps from -70 mV of VARNAM.A122D, with VARNAM as control. Number in parentheses indicates the number of neurons assayed. Values represent mean ± s.e.m. f. Fluorescence response to voltage steps of Positron$_{525}$-ST, compared to Positron$_{525}$.A122D-ST. Number in parentheses indicates the number of neurons assayed. Values represent mean ± s.e.m.

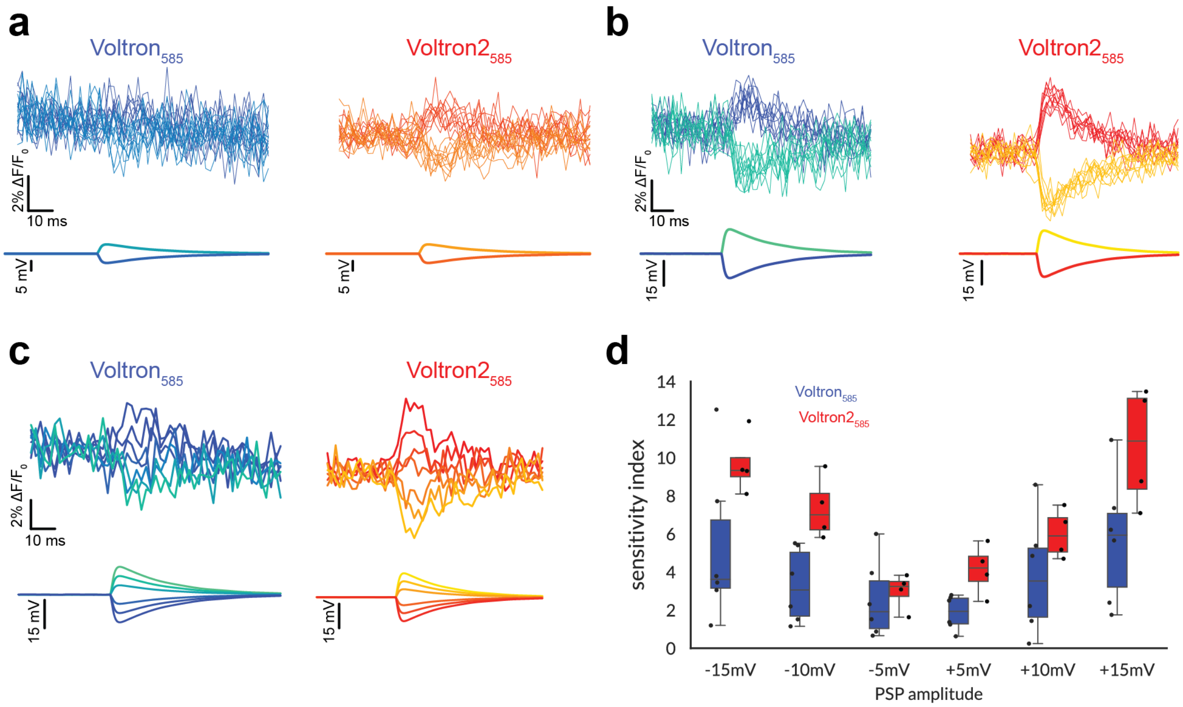

Figure S6. Individual trials and sensitivity in response to synPSPs in mouse brain slices. Related to Fig. 3

a,b. Representative fluorescence traces of $Voltron_{585}$ and $Voltron2_{585}$-expressing cells (n=10 trials from single cell) in response to ±5mV (a) and ±15mV (b) synPSPs. c. Representative traces of single trials in response to synPSPs ranging from -15mV to +15mV in 5mV steps. Traces from a single Voltron- and Voltron2-expressing cell are shown. d. Sensitivty index (d') for synPSPs in cells expressing Voltron and Voltron2; each point represents an individual cell.

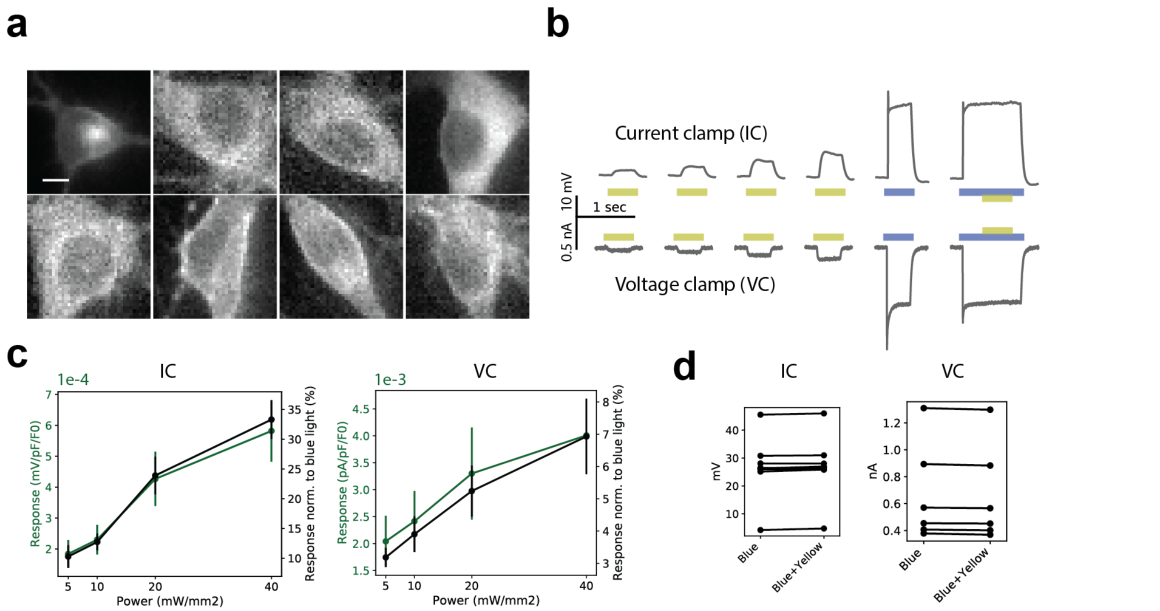

Figure S7. Yellow light evoked response in cultured hippocampal neurons expressing ACAGW-ChR2-Venus under voltage clamp (VC) and current clamp (IC). Related to Fig. 4.

a. Representative cells expressing ChR2-Venus. Scale bar: 5 μm. b. Yellow light evoked membrane voltages (top) and photocurrents (bottom) at 5, 10, 20 and 40 mW/mm^2 (575/25 nm, 400 ms) followed by blue light (470/24 nm, 50 mW/mm^2, 400 ms) and blue light (50 mW/mm^2, 1 sec) concurrent with yellow light (50 mW/mm^2, 400 ms). c. Yellow light-evoked membrane voltage (left) and current (right) normalized to membrane capacitance x F_0 and blue light response (mean ± s.e.m.). d. Effect of concurrent blue and yellow irradiation. Yellow light has no significant effect on blue light response (IC: p = 0.972; VC: p=0.957, Student's t test).

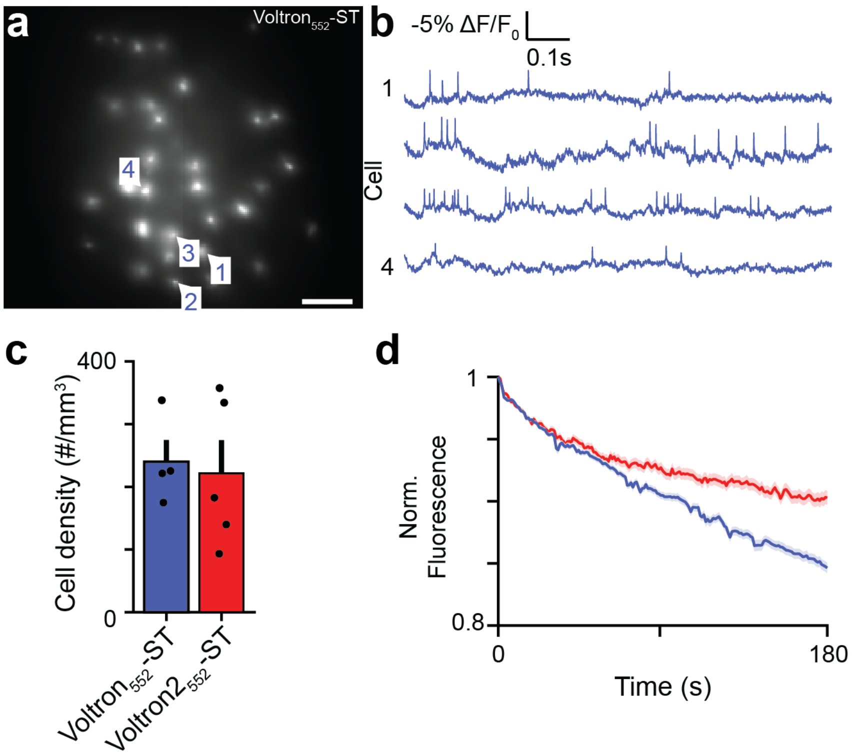

Figure S8. Voltron and Voltron2 imaging *in vivo* in mouse hippocampus. Related to Fig. 7.

a. Example image of hippocampal PV neurons expressing $Voltron_{552}$-ST. b. Sample fluorescence traces of cells 1-4 in (a). Scale bar: 200 µm. c. Density of visually identifiable neurons in mouse CA1. d. Photobleaching comparison of $Voltron_{552}$ (red) and $Voltron2_{552}$ (blue) in mouse CA1 (solid lines: mean, shading: s.e.m.); n=105 neurons, 7 imaging sessions.
